# Comparative genomics of human and non-human *Listeria monocytogenes* sequence type 121 strains

**DOI:** 10.1371/journal.pone.0176857

**Published:** 2017-05-04

**Authors:** Kathrin Rychli, Eva M. Wagner, Luminita Ciolacu, Andreas Zaiser, Taurai Tasara, Martin Wagner, Stephan Schmitz-Esser

**Affiliations:** 1 Institute for Milk Hygiene, University of Veterinary Medicine Vienna, Wien, Austria; 2 Vetsuisse Faculty, Institute for Food Safety and Hygiene, University of Zurich, Zurich, Switzerland; University of Copenhagen, DENMARK

## Abstract

The food-borne pathogen *Listeria (L*.*) monocytogenes* is able to survive for months and even years in food production environments. Strains belonging to sequence type (ST)121 are particularly found to be abundant and to persist in food and food production environments. To elucidate genetic determinants characteristic for *L*. *monocytogenes* ST121, we sequenced the genomes of 14 ST121 strains and compared them with currently available *L*. *monocytogenes* ST121 genomes. In total, we analyzed 70 ST121 genomes deriving from 16 different countries, different years of isolation, and different origins—including food, animal and human ST121 isolates. All ST121 genomes show a high degree of conservation sharing at least 99.7% average nucleotide identity. The main differences between the strains were found in prophage content and prophage conservation. We also detected distinct highly conserved subtypes of prophages inserted at the same genomic locus. While some of the prophages showed more than 99.9% similarity between strains from different sources and years, other prophages showed a higher level of diversity. 81.4% of the strains harbored virtually identical plasmids. 97.1% of the ST121 strains contain a truncated *internalin A* (*inlA*) gene. Only one of the seven human ST121 isolates encodes a full-length *inlA* gene, illustrating the need of better understanding their survival and virulence mechanisms.

## Introduction

*Listeria* (*L*.) *monocytogenes* is a well-studied food-borne pathogen known for causing listeriosis, a rare but severe infectious disease [1]. *L*. *monocytogenes* strains are often found in food and food production environments. Among those *L*. *monocytogenes* strains, particular strains belonging to sequence type (ST) 121 are highly abundant [2–12]. However, the molecular mechanisms responsible for the abundance of ST121 *L*. *monocytogenes* strains are still largely unknown. Despite a high number of available *L*. *monocytogenes* genome sequences, only a few studies have focused on genome analyses of *L*. *monocytogenes* ST121 genomes. Holch and coworkers analyzed the genomes of two persistent ST121 strains from Denmark and found almost no changes within the genomes of these persistent strains over a period of six years [13]. Ortiz et al. determined the genomes of four persistent ST121 strains and revealed differences in prophage regions [14]. In a recent study we performed genome analysis of nine ST121 *L*. *monocytogenes* strains, among them five persistent strains [15]. We identified highly conserved mobile genetic elements such as plasmids and some prophages. Additionally, we identified a number of candidate genes possibly involved in survival of ST121 *L*. *monocytogenes* strains in food and food production environments; like the transposon Tn*6188*, which confers increased tolerance towards various quaternary ammonium compounds, as we described recently [16, 17].

As the number of genomes analyzed (n = 9) in our last study was relatively low, we decided to perform a follow-up study of *L*. *monocytogenes* ST121 genomes using a much larger dataset. We have additionally sequenced 14 ST121 genomes and compared them to all currently available genomes (n = 56) of ST121 strains including seven human isolates. ST121 isolates (clonal complex CC121) are known to harbor a truncated *internalin A* (*inlA*) gene and have been described as hypovirulent in a humanized mouse model compared to isolates from CC 1, 4, and 6 [7]. Nevertheless, an occurrence of human ST121 *L*. *monocytogenes* isolates—although at a low incidence—has been described during the last years [4, 5, 7, 11, 18, 19]. Our genome data of this study now provides a high coverage of ST121 genomic diversity, allowing better comparative genome analyses focusing on survival mechanisms in food and food production environments, as well as virulence mechanisms of this highly abundant *L*. *monocytogenes* sequence type.

## Materials and methods

### DNA isolation and genome sequencing of *Listeria monocytogenes* ST121 strains

Fourteen ST121 *L*. *monocytogenes* strains were selected for genome sequencing (Table 1, for more details see S1 Table). These strains were comprised of isolates from food, food production environment, as well as three human isolates. *L*. *monocytogenes* strains were cultivated under aerobic conditions at 37°C in brain heart infusion broth (BHI, Merck; with 125 rpm shaking), harvested by centrifugation, the resulting pellet was used for DNA isolation using the NucleoSpin^®^ Tissue Kit (Macherery-Nagel) according to the recommendations of the manufacturer. Genome sequencing was performed with Illumina MiSeq sequencing technology using 300 bp read length and paired-end sequencing (Microsynth, Balgach, Switzerland). Between two and three million reads were used for a *de novo* assembly using ABySS with k-mer size of 64 [20]. The average coverage of the assembled contigs (> 500bp) ranged from 101x to 286x, while the number of contigs (> 500bp) per genome varied from 20 to 39.

**Table 1:** Origin of the ST121 strains sequenced in this study. For more details see S1 Table.

Strain	Year of isolation	Source	Country of origin
**AB27**	2013	food producing environment (floor)	Romania
**ABS6**	2013	food producing environment (meat grinder)	Romania
**L58-55**	2002	human	Austria
**N12-0367**	2012	human	Switzerland
**N13-0119**	2013	human	Switzerland
**P01-012**	2013	food (beef sausage)	Turkey
**P01-015**	2013	food (raw fish)	Turkey
**P02-001**	2012	food (sausage)	Russia
**P02-003**	2013	food (bacon)	Turkey
**P02-008**	2013	food (eggs)	China
**P02-011**	2013	food (sausage)	Russia
**P04-001**	2012	food (falafel powder)	Egypt
**Ro10**	2013	food (poultry)	Rep. of Moldavia
**Ro11**	2013	food (butter)	Rep. of Moldavia

### Comparative genome analyses

Automatic genome analysis and annotation of the genomes were done using the RAST server (http://rast.nmpdr.org/) [21]. Genome comparisons and determination of homologous proteins were done with BlastP, BlastN and tBlastN [22]. Similarly to a previous study [23], we used a similarity cut-off of 60% amino acid identity and 80% coverage for identification of homologous proteins. Alignments of plasmids were done with MAUVE [24]. Amino acid-based alignments were performed with MAFFT [25] and visualized with BOXSHADE (http://www.ch.embnet.org/software/BOX_form.html). The similarity of prophages was determined by BlastN searches using complete prophage sequences as query. Multilocus sequence typing (MLST) of the sequenced strains was performed with the MLST tool available on the Center for Genomic Epidemiology website (https://cge.cbs.dtu.dk/services/MLST/ [26]). For comparison, 56 publicly available ST121 genomes comprising food, food production environment, animal and human isolates were included in our analyses (See S1 Table for details). If draft genomes were available in GenBank, the contigs were uploaded into the RAST server for annotation. For some genomes, only raw sequencing reads were deposited in GenBank sequencing read archive SRA; for these genomes, assemblies were performed as described above.

To evaluate the similarity between the 70 ST121 genomes, we determined the average nucleotide identity (ANI) based on MUMmer and the correlation indexes of tetra nucleotide signatures (tetra) [27] using the JSpeciesWS webserver (S2 Table) (http://jspecies.ribohost.com/jspeciesws/ [28]). To visualize the similarity between the ST121 genomes, we used Orange 3.3 [29]. To create a distance file, we subtracted the correlation indexes of tetra nucleotide signatures (tetra) from 1 and multiplied by 1000. Multidimensional scaling constructed from distance matrix (MDS) (maximum iteration 3000, PCA (Torgerson), no jitter) was used for visualization. The differences between each of the genomes represent relative values.

### Virulence assays

For the *in vitro* virulence assay, human intestinal epithelial (Caco2, ATCC^®^ HTB-37^™^) and hepatocytic (HepG2, ATCC^®^ 8065^™^) cells were cultivated in Eagle’s minimum essential medium (MEM, Fisher Scientific) containing 2 mM L-glutamine, 10% fetal bovine serum (FBS), 100 units/ml penicillin, 100 mg/ml streptomycin sulphate and 0.25 mg/ml amphotericin B at 37°C in a humidified atmosphere (95% relative humidity) containing 5% CO_2_. One colony of *L*. *monocytogenes* was inoculated in brain heart infusion complemented with yeast extract (BHI-Y, Merck) and cultivated for 8 h at 37°C with shaking (120 rpm). The bacterial culture was adjusted to OD_600_ 0.1 in 5 ml BHI-Y and grown for 16 h at 10°C without shaking mimicking natural contamination conditions. Cell monolayers were infected with *L*. *monocytogenes* at a multiplicity of infection of 25 for 1 h at 37°C. The cell monolayers were washed with Dulbecco’s Phosphate Buffered Saline (PBS; Fisher Scientific) and incubated in MEM, 10% FBS containing gentamicin (100 μg/ml) for 45 min (invasion) and 4 h (intracellular growth), respectively. The cells were lysed with 1 ml 0.1% Triton X-100 (Merck) and colony forming units (CFU) were determined by plating on tryptic soy agar complemented with yeast. The invasion efficiency (%) was calculated as mean CFU recovered after 45 min of gentamicin treatment divided by CFU of the inoculum. The intracellular growth coefficient (IGC) was calculated as follows: IGC = (intracellular bacteria_4h_-intracellular bacteria_45min_)/ intracellular bacteria_45min_. Each experiment was performed in triplicate and repeated at least 3 times. Two reference strains EGDe and ScottA were included in the *in vitro* virulence assays.

Microsoft Excel^®^ 2007 and SPSS.20 software (SPSS Inc., Chicago USA) were used for statistical analysis. Brown Forsythe and Welch test were used to confirm the variance homogeneity, and posthoc test (Tukey-HSD) was used to determine significant differences between the strains (P< 0.05).

### Availability of data

The genome and plasmid sequences of the 14 strains sequenced in this study have been deposited in DDBJ/EMBL/GenBank under BioProject accession number PRJNA335730.

## Results and discussion

### General features of *L*. *monocytogenes* ST121 genomes

**Fig 1:**
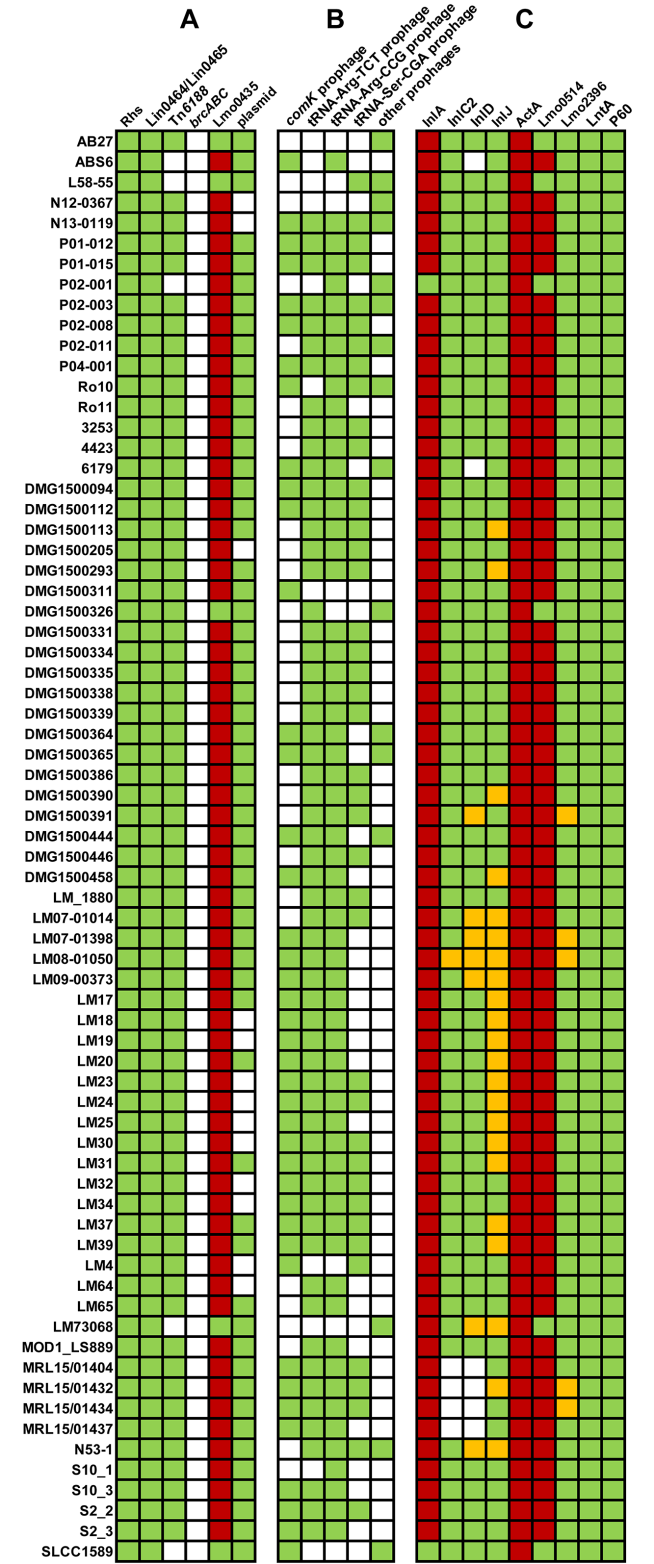
Distribution of selected genetic elements among *L*. *monocytogenes* ST121 genomes. The columns show the presence (colored)/absence (white) and full-length (green) or truncation (red) of ST121 genetic features including factors possibly involved in adaptation to survival in food production environments (**A**), selected prophages (**B**) and virulence factors (**C**). Yellow indicates incomplete sequence for the respective feature.

In this study we analyzed and compared the genomes of 70 *L*. *monocytogenes* ST121 strains: 14 *L*. *monocytogenes* ST121 strain genomes were sequenced and 56 *L*. *monocytogenes* ST121 genomes were retrieved from GenBank. This included strains from 16 different countries from different isolation years deriving from the following sources: food (n = 39), environment (n = 21), animal (n = 2) and human (n = 7). The source of one strain is unknown. While some of the isolated strains have been described to be persistent [8, 13–15], we do not have information on persistence of the other ST121 strains in our study. Details on the strains and genomes can be found in S1 Table. The *L*. *monocytogenes* ST121 genomes have assembly sizes ranging from 2.95 to 3.23 Mbp. The differences in assembly sizes are mostly due to the presence or absence of plasmids—which are present in 81.4% of all strains—and of prophage regions (Fig 1, S2 Table). The total number of prophages per strain varied from zero to six.

**Fig 2:**
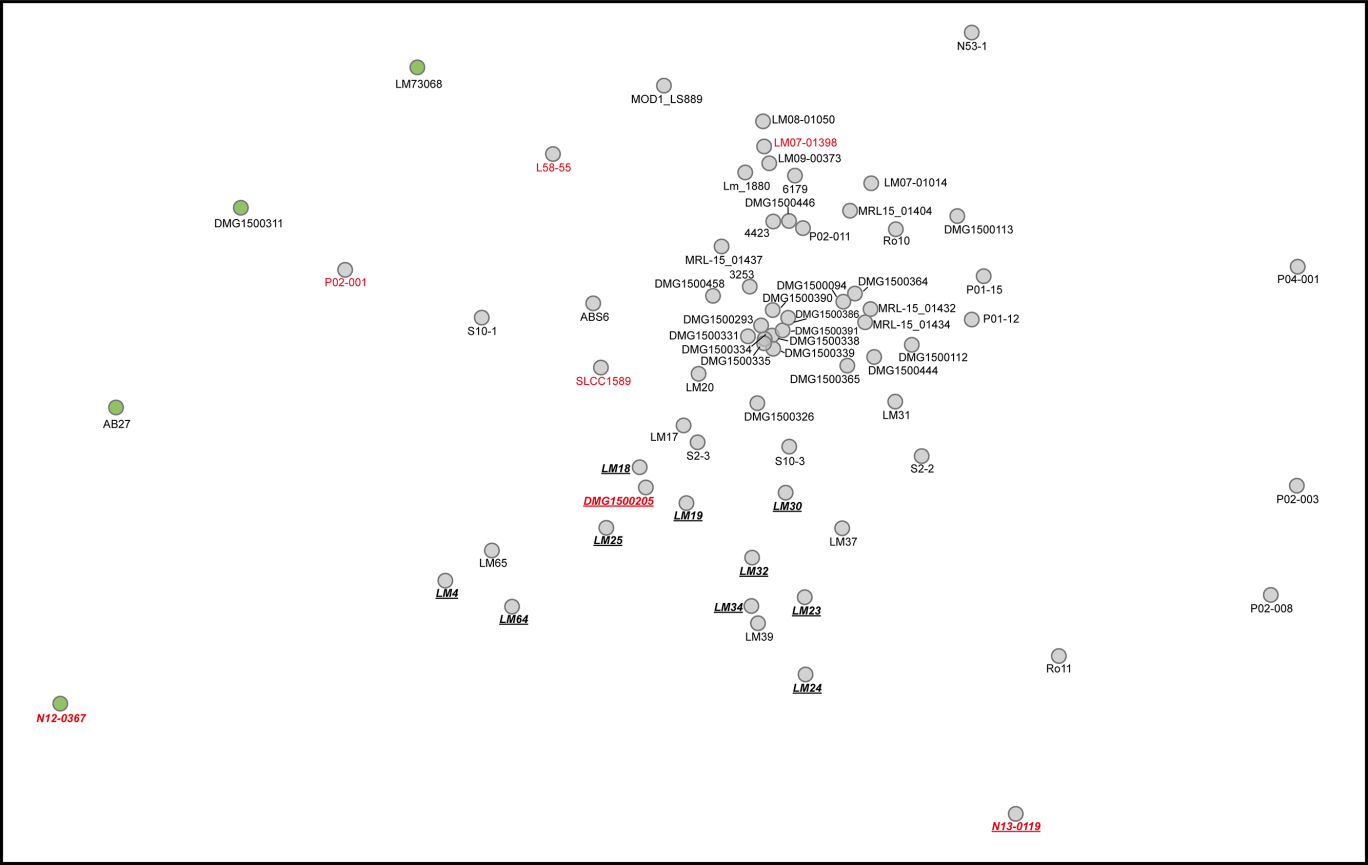
Similarities between the 70 *L*. *monocytogenes* ST121 genomes revealed by tetranucleotide signature correlations. The correlation indexes of tetra nucleotide signatures (tetra) based on whole genome sequences was calculated using JSpeciesWS [28]. Visualization was performed creating a multidimensional scaling constructed from distance matrices (MDS) using Orange 3.3 [29]. The shorter the distance between the points, the higher the similarity based on whole genome sequences. Green: strains harboring ≤ 1 prophage, red label: human isolates, italic-bold underlined label: strains devoid of plasmids. The differences between each of the genomes represent relative values.

The ST121 genomes are highly conserved and share more than 99.73% average nucleotide identity (ANI, S3 Table). To visualize the similarities between the 70 ST121 genomes, we calculated a distance matrix based on whole genome sequences and correlation indexes of tetra nucleotide signatures (Fig 2). The differences between the strains were mainly based on the absence/presence of plasmids and the number of prophages (e.g. strains devoid of plasmids clustered). We detected a high similarity between the strains isolated from Australia (all but four clustered) and between the four strains isolated in France. The seven human isolates did not cluster. The recently described nine hypervariable hotspots [23] showed identical gene content among the ST121 strains.

### Genes possibly associated with survival in food and food production environments

As ST121 strains are often found to be persistent in food production environments, we analyzed the ST121 genomes for the presence of genes possibly involved in survival in food production environments (Fig 1, S2 Table). 92.8% of the ST121 strains harbored the transposon Tn*6188*, which we characterized recently to be responsible for increased tolerance against various quaternary ammonium compounds [16, 17]. The Tn*6188* copies show more than 99.99% nucleotide identity to each other. The presence of Tn*6188* is not restricted to ST121 strains. However, Tn*6188* is particularly abundant among ST121 strains, which is in line with other recent studies [3, 12, 14, 16, 30]. The *bcrABC* cassette, another mostly plasmid-borne genetic feature responsible for tolerance against quaternary ammonium compounds [31, 32], was absent from all ST121 genomes. All ST121 strains harbored the previously described [33] insertion of *L*. *innocua lin0464/lin0465* genes between *lmo0443* and *lmo0449* homologues (the insertions of all strains show 100% amino acid identity to each other). Strains devoid of *lin0464*/*lin0465* harbor either the stress survival islet-1 (SSI-1), linked to tolerance towards acidic, salt, bile and gastric stress [34–36] or a homologue of the uncharacterized *LMOf2365_0481* gene. Preliminary data suggests that Lin0464, a putative transcriptional regulator, and Lin0465, an intracellular pfpI protease are involved in stress survival (unpublished data).

Recently, we identified a novel 12.1 kb insert in ST121 genomes harboring—among others—a protein with RHS domains and a putative RNA 2’-phosphotransferase (KptA) [15]. This insert was present in all ST121 genomes and is almost identical among all ST121 genomes (>99.99% nucleotide identity). Since RHS proteins have been shown to be involved in competition against other bacteria as shown e.g. in *Bacillus subtilis* and *Serratia marcescens* [37, 38], we thus assume that the RHS protein region in ST121 strains has a similar function.

Furthermore, 91.4% of the ST121 strains harbor a truncated *lmo0435* (bapL) gene, an LPXTG protein which has been suggested to be involved in attachment to abiotic surfaces [39]. Interestingly, all strains harboring a truncated *lmo0435* gene, show the identical N-terminal truncation resulting in two predicted proteins with 60 and 1825 amino acids length compared to the 2013 amino acid full-length protein (S1 Fig). This N-terminal truncation results in the loss of the predicted signal peptide. The occurrence of identical truncations in such a high percentage of otherwise unrelated ST121 strains is surprising, as an internal stop codon resulting in a truncated version of a protein should render it non-functional and result in the accumulation of deleterious mutations due to the lack of evolutionary pressure. We therefore hypothesize that the ST121 *lmo0435* homologues harboring predicted internal truncations might be functional despite the truncations and have additional, yet unknown functions which might not require a signal peptide. It should be noted that *lmo0435* is not present in all *L*. *monocytogenes* genomes. An alternative explanation for the occurrence of identical truncations might thus be that strains harboring the same truncations might derive from the same recent common ancestor. The presence of full-length and truncated *lmo0435* homologues indicates that the truncated *lmo0435* variants arose independently in a subset of ST121 strains.

### Prophage content and conservation

**Fig 3:**
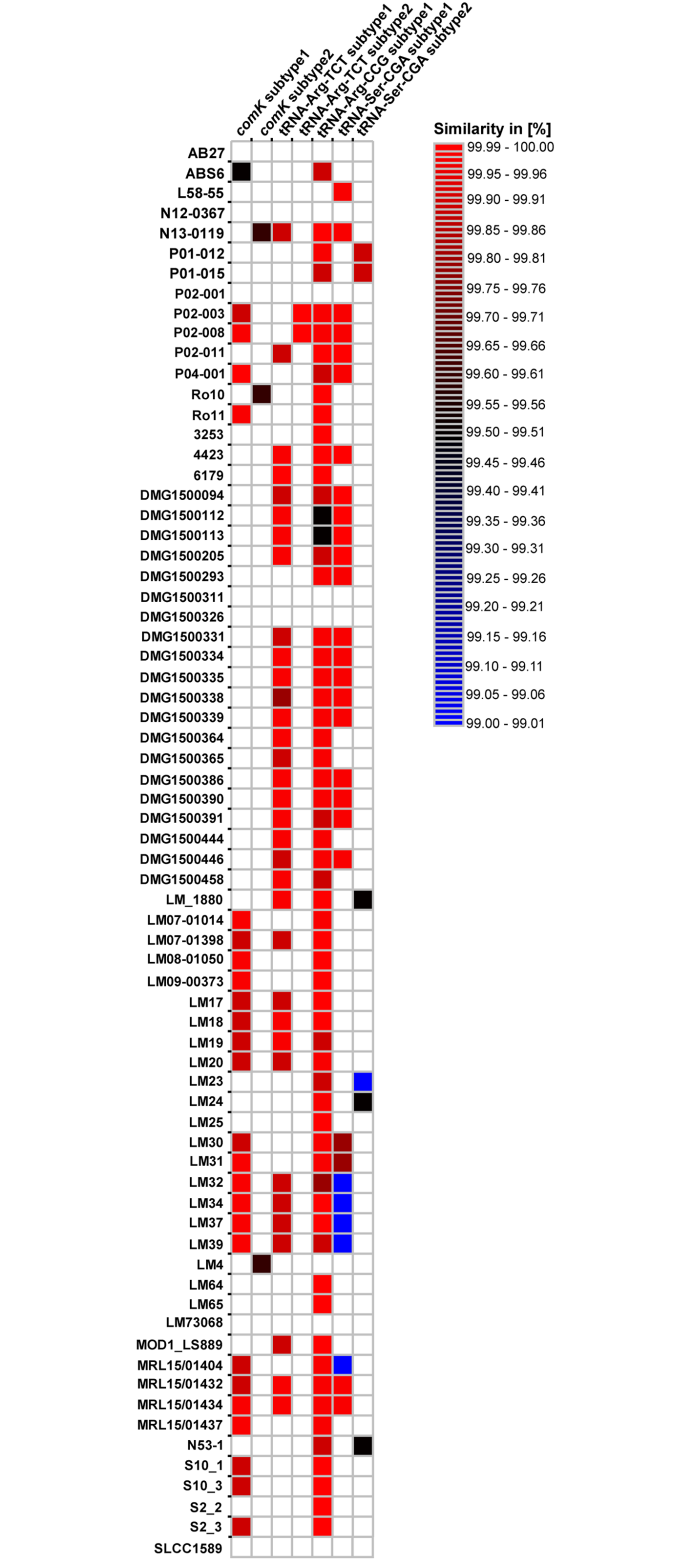
Similarities of selected *L*. *monocytogenes* ST121 prophages. Heatmap showing the presence/absence and conservation (% identity on DNA level) between prophages of the same subtype of selected ST121 prophages. Only prophages with more than 99% similarity to each other within their respective subtypes are shown.

In our previous study on *L*. *monocytogenes* ST121 strains, we detected an extraordinarily high degree of conservation among some of the prophages found in ST121 genomes [15]. Here, using a much larger dataset of 70 genomes, we could to a large extent confirm our previous results and present also a number of novel findings. We first analyzed the presence/absence of prophages inserted at the same genomic loci (Figs 1 and 3, S2 Table).

In total, four abundant prophage insertion sites were found in ST121 genomes: 58.6% harbor a prophage inserted downstream of the Ser-CGA tRNA, 61.4% a prophage inserted into the *comK* gene, 84.3% a prophage inserted downstream of the Arg-TCT tRNA, and 88.6% of the strains harbor a prophage inserted downstream of the Arg-CCG tRNA. In addition to these abundant prophage insertions, we also detected less abundant prophage insertions in different genomic loci of ST121 strains. We found an insertion of prophages at the Thr-GGT tRNA in 7.1% of the strains, which has been described previously [23]. Furthermore, prophages were inserted between the *lmo1702* and the *trmA* gene (*lmo1703*) in 5.7% of ST121 strains as described for other *L*. *monocytogenes* ST recently [40–42]. The region between the *lmo1702* and *lmo1703* homologues in ST121 strains has a size of approximately 50 kbp and a GC content of 41.9% and contains one prophage (size approximately 36.2 kbp) which shows highest similarity to *Listeria* phages and phages from *Lactococcus lactis* A12. In addition, this region also encodes a number (n = 16) of proteins showing no or only low similarity to *Listeria* including two proteins representing a putative HpaI-like type II restriction system, which has not been described in *Listeria* before. Based on the definition by Kuenne et al. [23], the *lmo1702*/*lmo1703* locus represents a novel hypervariable hotspot. Two ST121 strains (N12-0367 and Ro11) harbor a highly similar (93.7% identity, 43.7 kb overlap) insertion between *lmo1750* and *lmo1751* homologues. The *lmo1750/lmo1751* inserts of N12-0367 and Ro11 harbor the same novel putative HpaI-like restriction system mentioned above. Of note, the *lmo1702*/*lmo1703* insert of N13-0119 (a human isolate from Switzerland, 2013) and the *lmo1750*/*lmo1751* insert in Ro11 (a 2013 food isolate from Moldavia) are identical. We also identified another novel region harboring a prophage, which is inserted into the *lmo1263* homologue—a putative transcriptional regulator—in 7.1% of the strains. This prophage shows highest similarity to other *Listeria* phages. In addition, we also identified a prophage inserted between the *lmo0271* and *lmo0272* genes in one strain (P04-001).

Apart from the presence/absence of prophages, we were also interested in the degree of conservation of prophages. We have previously shown that the tRNA-Arg-CCG and tRNA-Arg-TCT prophages are specific to ST121 *L*. *monocytogenes* and show a very high degree of conservation, although these strains derive from different origins and years [15]. Similar results have been described recently for *L*. *monocytogenes* ST8 strains, in which the authors found an extremely high degree of conservation of prophages within the same subgroup, but higher diversity of prophages between subgroups [43]. Also ST204 *L*. *monocytogenes* strains showed highly conserved prophages, however to a lesser degree as seen in ST121 [44].

The prophages inserted downstream of the Ser-CGA tRNA reveal the presence of two highly conserved but distinct subtypes: subtype 1 is found in 28 strains harboring a Ser-CGA tRNA prophage, subtype 2 is found in seven strains (Fig 3). The conservation within each subtype is higher than 99.5%. Subtype 1 and subtype 2 share 95% similarity with approximately 83% coverage.

It has been suggested that prophages inserted into the *comK* gene are important for survival in food production environments [45]. However, it should be noted that the *comK* prophages in ST121 strains show only low similarity to the *comK* prophages described by Verghese and coworkers [45]. The *comK* prophage harboring strains (n = 43) could be divided in two groups: 24 contain the *comK* subtype 1, whereas three contain a different prophage (*comK* subtype 2), which shows 91% similarity with approximately 50% coverage to the subtype 1 *comK* prophage. Within each of the *comK* prophage subtypes, the conservation of the prophages is higher than 99.5%. The *comK* prophages of the other 16 strains are distinct.

Similar to the aforementioned prophages, different subtypes were also found within the tRNA-Arg-TCT prophage: the most abundant subtype, which is present in 33 of the strains, is represented by the tRNA-Arg-TCT prophage identified previously in strain 6179. The conservation within this subtype is higher than 99.9% similarity, a second related, but distinct subtype is found in two strains (P02-003 and P02-008). The two subtypes show 93% similarity to each other with approximately 77% coverage.

The 6179-like tRNA-Arg-CCG prophage subtype was found in 58 out of 62 strains, with a high conservation within this subtype (>99.9% similarity).

Three of the four inserts between *lmo1702* and *lmo1703* show more than 99.9% similarity, whereas the insert in strain AB27 shows only 90% similarity to the other inserts. Furthermore, all five prophages inserted into the *lmo1263* gene, show 99.9% similarity to each other.

A beneficial role of prophages in stress survival has been described by a number of studies [45–48]. An induction of prophage gene expression after acid stress exposure has been recently reported for the *L*. *monocytogenes* 10403S A118 prophage and the *lmaDCBA* operon [49]. In contrast, downregulation of prophage gene expression was described for the ST121 strain 6179 under sublethal quaternary ammonium compound stress conditions [50]. Prophage induction in mixed populations might facilitate horizontal gene transfer, allowing the acquisition of novel genetic material. In addition, prophage induction might provide an advantage, mediating bacteria-bacteria competition by killing or inhibiting other strains in food production environments or during mixed infections [51–53]. We have currently no evidence whether the ST121 prophages might be induced to form lytic phage particles under conditions in food production environments.

### Plasmids in ST121 strains

In our previous study we found plasmids in all nine analyzed ST121 genomes. Here, with our larger dataset (n = 70), we detected plasmids in 81.4% of all strains. While the vast majority of strains harbor the same plasmid sharing more than 99.9% sequence similarity to pLM6179, which has a size of 62.2 kbp, [15], strain P02-001 harbors a plasmid with a size of approximately 59 kbp which is most similar to the 148 kbp plasmid from *L*. *monocytogenes* N1-011A (99% identity and coverage; S2 Fig). In some strains derived from a study from Italy [8], we were not able to unambiguously identify all plasmid contigs due to the high number of relatively small contigs in the assemblies. All identified plasmid contigs in those strains show > 99.9% similarity to pLM6179. However, we cannot exclude that some of these plasmids could be different from the pLM6179-like plasmids.

We detected a lower percentage of ST121 strains harboring plasmids in our current study compared to our previous study (81.4% and 100%). This might be explained by either the absence of plasmids in some strains, or the loss of plasmids during isolation and cultivation. Nevertheless, the vast majority of ST121 strains carrying a plasmid harbor the same highly conserved pLM6179-like plasmids. Similarly, 86% of ST204 *L*. *monocytogenes* strains were found to harbor plasmids, although their plasmids showed higher diversity compared to those in ST121 [44]. We hypothesize that the ST121 plasmids confer advantages during stress conditions. Previous work has shown that the plasmid-encoded cadmium resistance determinant Tn*5422*, which is present on all ST121 plasmids, provides resistance towards cadmium [54–56]. A recent study has suggested that the Tn*5422* cadmium resistance locus also provides higher tolerance towards bacteriophages, although the mechanisms responsible for phage tolerance are still unknown [57]. Although plasmids are common among *Listeria* strains [44, 58], and plasmids generally provide advantages to their hosts, the knowledge about the function of *Listeria* plasmids is still very limited.

### Insertion of a *Lactococcus*-derived LlaI restriction modification system locus in strain AB27

The *lmo1702/lmo1703* insert in strain AB27 shows only approximately 90% similarity to the *lmo1702/lmo1703* inserts found in other strains analyzed in this study. In AB27, this includes a 6.8 kbp region showing 99.7% similarity to the LlaI restriction modification system found on *Lactococcus lactis* plasmid pTR2030 which confers phage resistance [59]. The presence of the LlaI restriction modification system in AB27 might thus explain the absence of additional prophages in AB27.

### Virulence genes and potential of ST121 *L*. *monocytogenes* strains

*L*. *monocytogenes* of ST121 are among the most prevalent clones in food, but are underrepresented in clinical samples [4, 5, 7, 11, 12, 18, 19]. Among the 70 ST121 strains analyzed in our study, seven strains were human isolates. The virulence of ST121 strains is known to be attenuated primarily due a truncation in the *inlA* gene [7, 60–62], which has so far been reported for all ST121 strains. Two ST121 strains of our study encode a full-length InlA, among them one human isolate. All other ST121 strains harbor an identically truncated InlA (mutation type 6) with a predicted length of 491 amino acids [61, 62]. In addition, we analyzed the presence and conservation of 85 virulence-associated genes including internalins and internalin-like proteins using BlastP and tBlastN, based on den Bakker and coworkers [63] (Table 2). All ST121 proteins with an amino acid identity lower than 99% to EGDe were analyzed in more detail. All of the 85 analyzed virulence genes present in EGDe [63], except homologs of *lmo1099*, *lmo1102* and *lmo2026*, are present in the ST121 genomes (data not shown). Overall, all ST121 strains encode a highly similar set of virulence genes with high amino acid identity to their EGDe homologues.

**Table 2:** Presence/absence and similarity of selected virulence factors in ST121 and other *L*. *monocytogenes* strains.

		ST121 strains				EGDe		ScottA		10403S		F2365	
protein	function	type of variation in ST121 strains	length (amino acids)	presence of homologue in % of ST121 strains ^a^	incomplete sequence	% amino acid identity to strain 4423	length (amino acids)	% amino acid identity to strain 4423	length (amino acids)	% amino acid identity to strain 4423	length (amino acids)	% amino acid identity to strain 4423	length (amino acids)
**InlA**	invasion	truncation (n = 68)	491	95.71% (n = 67)	1.43% (n = 1)	98.98	800	98.17	800	98.78	800	98.17	800
**InlC2**	*in vivo* infection		548	88.57% (n = 62)	1.43% (n = 1)	-		96.90	548	94.71	548	96.72	548
**InlD**			567	78.57% (n = 55)	10.00% (n = 7)	-		89.61	568	96.65	567	89.61	568
**InlJ**	*in vivo* infection	long variant (n = 45)	921	68.18% (n = 45)	31.82% (n = 21)	84.26	794	-		-		93.92	921
**ActA**	cell-to-cell spread	internal truncation (n = 70)	604	100% (n = 70)	-	92.17	639	92.14	598	90.84	633	91.87	603
**LntA**	host immune response		211	100% (n = 70)	-	91.22	205	94.31	211	92.89	211	93.23	192
**Lmo0514**	*in vivo* infection	truncation (n = 64)	549	91.43% (n = 64)	-	97.81	605	96.51	609	99.10	611	96.51	609
**Lmo2396**	unknown		872	85.71% (n = 60)	7.14% (n = 5)	80.23	940	86.49	681	83.83	872	84.68	795
**P60**	cell-to-cell spread		476	98.57% (n = 69)	-	98.55	482	97.69	477	98.74	476	97.06	477

^a^ defined as presence of homologues with 100% amino acid identity to strain 4423.

The *L*. *monocytogenes* pathogenicity island 1 (LIPI-1) of all 70 analyzed strains includes an actin-assembly inducing protein (ActA) variant with an internal deletion resulting in a length of 604 amino acids, which has been reported for ST121 strains recently [7]. This ActA variant shares 92.17% amino acid identity to ActA of EGDe (S3 Fig). ActA is responsible for bacterial movement and cell-to-cell spread and has recently been shown to also be involved in biofilm formation and aggregation as well as long-term colonization of the gut [64]. However, studies on the effect of different ActA variants on the virulence potential of *L*. *monocytogenes* show inconsistent results [65, 66].

The LPXTG protein InlJ harbored by the ST121 strains consists of 921 amino acids (including 5 mucin-binding protein (MucBP) domains) and shows only 84.26% amino acid identity to InlJ of EGDe (794 amino acids and 4 MucBP domains, S3 Fig). A full length *InlJ* gene was identified in 45 strains (68.18%) only. However, we found evidence for the presence of *InlJ* (based on the presence of highly similar contigs) in all ST121 strains. InlJ has an important role in *L*. *monocytogenes* infection and has been shown to act as an adhesin [67, 68]; however, whether the longer InlJ variant present in ST121 strains has an effect on bacteria-host interaction or virulence is currently unknown. While MucBP domains have been shown to be responsible for MUC2 binding (the major component of intestinal mucus), the MucBP domains of InlJ have been shown to be dispensable for MUC2 binding, suggesting that the MucBP domains in InlJ have a different function [69].

The LPXTG cell wall protein Lmo0514, recently identified to be highly abundant in intracellular *L*. *monocytogenes* and to be required for virulence in mice at early infection stages and for survival under low pH [70–72], is present in two variants. 91.4% of all ST121 strains harbor the same truncated Lmo0514 with a length of 549 amino acids, and six strains harbor a full-length variant with 611 amino acids, among them three human isolates (S3 Fig). The truncated Lmo0514 homologues lack the C-terminal LPXTG cell wall anchor domain which might contribute to reduced virulence of ST121 strains. Interestingly, we observed that all ST121 strains harboring a truncated Lmo0514 homolog also have a truncated Lmo0435 homolog, whereas all ST121 strains with a full-length Lmo0514 homolog also harbor a full-length Lmo0435 homolog. Similarly to what we discussed above, the occurrence of identical truncations in such a high percentage of otherwise unrelated ST121 strains is surprising, as an internal stop codon resulting in a truncated version of a protein should render it non-functional and result in the accumulation of deleterious mutations due to the lack of evolutionary pressure. It is thus tempting to speculate that Lmo0514 might have additional yet unknown functions which might be independent of the LPXTG cell wall anchoring domain. It might also be conceivable that Lmo0435 and Lmo0514 might be functionally linked. While we cannot exclude that the truncated Lmo0514 (and Lmo0435) homologues derive from a recent common ancestor and represent putative pseudogenes, the occurrence of identical mutations in unrelated *Listeria* strains has been reported previously [73, 74]. We hypothesize that the observed identical mutations might provide the ST121 strains with yet unknown selective advantages, possibly unrelated to virulence.

Another genomic region distinct from strain EGDe, but almost identical in all ST121 genomes (>99.7% similarity) is the region between the *lmo0061* and *lmo0075* homologues. This hypervariable hotspot 1 [23] harbors the putative type VII (T7SS, also called ESX-1, ESAT-6, WSS, or WXG100) secretion system. The region downstream of *esaD* (*lmo0066* homologue) is different in the ST121 genomes compared to EGDe (S4 Fig). The T7SS is well-described in *Staphylococcus aureus*, *Bacillus anthracis* and *Mycobacterium tuberculosis* [75]. A recent study showed that the T7SS is functional in EGDe but dispensable and even detrimental for virulence in EGDe and other *L*. *monocytogenes* [76]. A possible alternative function of the *L*. *monocytogenes* T7SS might be competition against other *Listeria* strains, as recently shown for the *Staphylococcus aureus* T7SS where EsaD—a Lmo0066 homolog—is a toxin targeting competitors [77].

Our study includes seven human ST121 *L*. *monocytogenes* isolates; all but one human isolate (SLCC1589), harbor a truncated *inlA* gene, known to be the main factor for invasion of intestinal epithelial cells. We performed *in vitro* virulence assays using strains grown to stationary growth phase at 10°C in rich media (mimicking the natural food contamination conditions) including three human isolates (L58-55, N12-0367, N13-0199; all harboring a truncated *inlA*), the food isolate 4423 (truncated *inlA*), strain P02-001 (food isolate, full length *inlA*) and two reference strains (EGDe and ScottA, both full length *inlA*) using human intestinal epithelial Caco2 and hepatocytic HepG2 cells.

**Fig 4:**
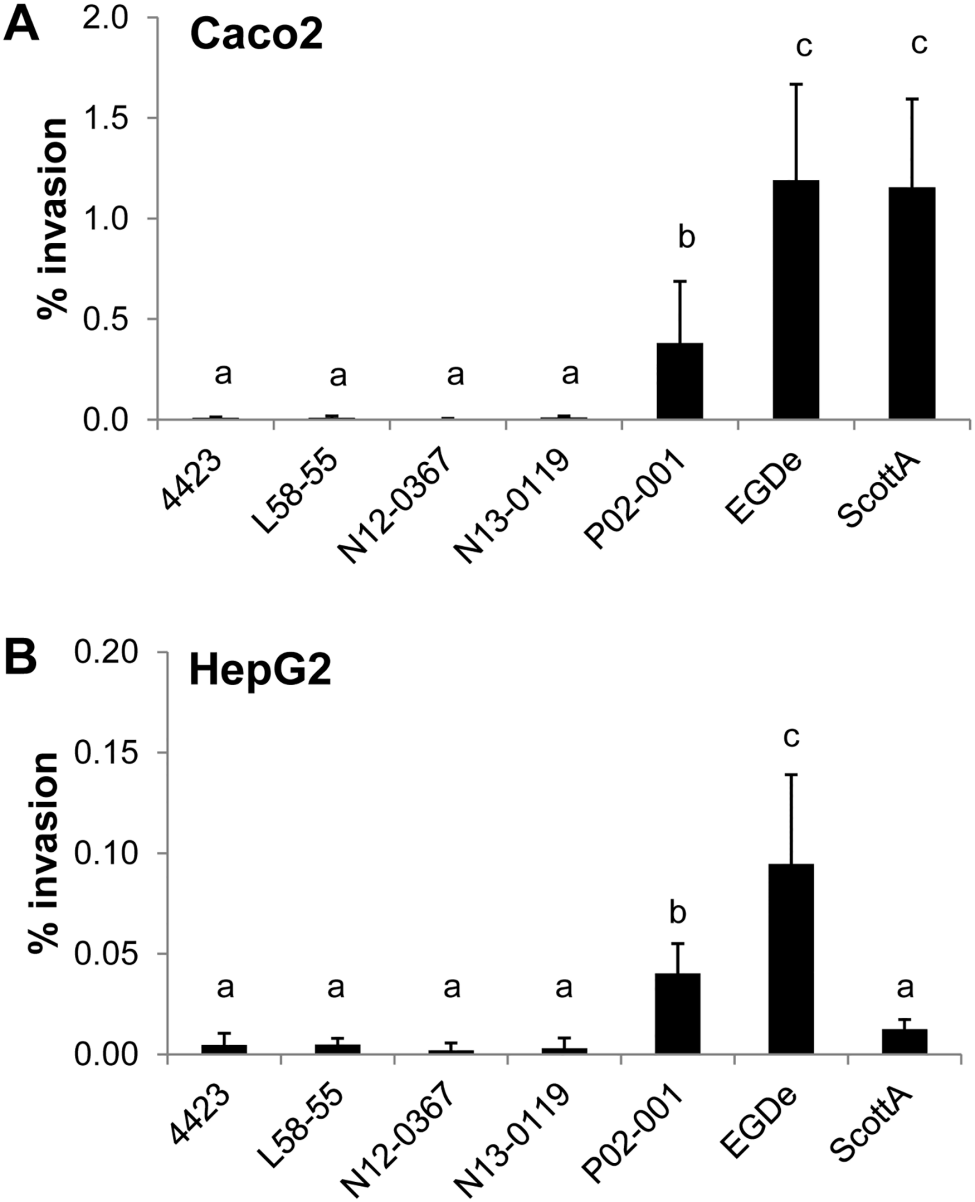
Invasion efficiency of *L*. *monocytogenes* ST121 strains in human cell lines. Invasion efficiency of three human ST121 isolates (L58-55, N12-0367, N13-0119; all harboring a truncated *inlA*), the food isolate 4423 (truncated *inlA*), strain P02-001 (food isolate, full length *inlA*) and the reference strains EGDe and ScottA in Caco2 (**A**) and HepG2 cell lines (**B**). Mean values and standard deviations of the three independent biological replicates are presented. Different letters indicate significant differences (P<0.05) between the invasion efficiency of the strains.

Invasion efficiency was significantly higher for strain P02-001 harboring a full length *inlA* compared to the ST121 strains with a truncated *inlA* but lower than the reference strain EGDe in both cell types (Fig 4). The second reference strain ScottA showed high invasion efficiency in Caco2 cells (comparable to strain EGDe) and a low invasion capability in HepG2 cells comparable to the ST121 strains harboring a truncated *inlA*. As expected, the effect of truncated *inlA* was more prominent using Caco2 than HepG2 cells. The differences in the intracellular growth between the strains were small. P02-001 showed a slightly lower intracellular growth compared to the other ST121 strains and EGDe in Caco2 cells and the intracellular growth of strain EGDe was slightly decreased in HepG2 cells (S5 Fig). These data suggest that all tested strains can replicate intracellularly once inside the host cell.

Although the majority of human ST121 isolates analyzed in this study (six out of seven) encode a truncated *inlA* gene and show attenuated invasion efficiency in human cells (shown here for three of the human ST121 isolates), they must have been able to infect humans and cause listeriosis. A recent study including in total 1167 ST121 strains—among them 82 human isolates (7%)–showed that most of the human ST121 isolates (79%) caused bacteremia; whereas 20% caused central nervous system infection and only one strain was isolated from a maternal-neonatal listeriosis [7]. This clearly shows the infection potential of ST121 strains, despite the presence of a truncated InlA. The invasion potential of ST121 strains harboring a truncated InlA into human cell lines is low, but a certain number of bacteria is nevertheless able to enter the host cell. Once in the host cell the ST121, strains are able to replicate intracellularly. In parallel, the colonization of the liver and spleen by ST121 isolates in orally infected mice was reduced [7], but still possible; this suggests that other virulence mechanisms exist in ST121 strains compensating for the missing InlA/E-cadherin interaction which is essential for invasion into intestinal epithelial cells. In line with this, clinical *L*. *monocytogenes* strains of other STs harboring truncated *inlA* genes have been described previously [62, 78–80]. These findings suggest that *inlA*-independent invasion pathways for certain tissues or organs may exist. This is supported by the finding that ST121 strains harboring truncated *inlA* genes are able to cross the placental barrier and infect fetuses in mice and guinea pigs [81].

Another reason enabling human listeriosis cases by ST121 strains, in spite of truncated *inlA* genes, might be the infectious dose and/or preconditions of the patients. Listeriosis caused by ST121 strains could be more prevalent in patients with a low health status, such as a dysfunctional intestinal epithelial barrier. Human listeriosis cases have been described for ST121 strains previously [4, 5, 7, 18, 19], although at a low incidence. Furthermore, a higher infectious dose of ST121 strains might be required to cause listeriosis. Nevertheless, the ability to cause listeriosis should be taken into account in risk assessment when dealing with *L*. *monocytogenes* strains harboring truncated *inlA* genes such as ST121 and ST9 [7, 12].

## Conclusions

Here we provide the first large-scale analyses of *L*. *monocytogenes* strains belonging to the same sequence type. We show that ST121 genomes are highly conserved and that variation between strains mostly occurs in prophage regions resulting in highly different genome sizes of ST121 strains. We also identified three novel prophage insertion sites. Interestingly, we identified similar, but distinct prophages inserted at the same genomic loci, with some prophage subtypes showing an extremely high degree of conservation between isolates from different origins. Prophages thus seem to be the main factor responsible for genomic differences among *L*. *monocytogenes* ST121 strains.

## Supporting information

S1 Fig Amino acid alignment of Lmo0435 homologues. Amino acid alignment of full-length and truncated Lmo0435 homologues in representative ST121 *L*. *monocytogenes* (AB27, full-length and 4423, truncated) compared to Lmo0435 from EGDe. The predicted signal peptides are highlighted in blue. For 4423, both predicted truncated Lmo0435 homologues are shown.
(PDF)

S2 Fig Alignment of selected ST121 plasmids. The plasmids were aligned using Mauve. Homologous regions are shown in the same color. The height of the similarity profile within each block corresponds to the average level of conservation in that region of the plasmids.
(PDF)

S3 Fig Alignments of ST121 virulence genes. Amino acid alignment of selected virulence factors in L. monocytogenes strains 4423 (ST121), EGDe, 10403S, ScottA, F2365. Internalin A (A), Internalin C2 (B), Internalin D (C), Internalin J (D), ActA (E), Lmo0514 (F), Lmo2396 (G), P60/Iap (H), LntA (I). The respective amino acid sequences of strain 4423 are shown as a representative for all other ST121 sequences except for the Lmo0514 (F), where also the Lmo0514 sequence from strain AB27 (a full-length sequence) is shown. The predicted C-terminal LPXTG cell wall anchoring domain in Lmo0514 is highlighted in blue (F). The key amino acids forming the binding motifs of LntA are highlighted in red (I). The ST121 sequences are highlighted in bold.
(PDF)

S4 Fig Organization of the WSS/type VII secretion system in *L*. *monocytogenes* EGDe and ST121 strains (represented by strain 6179). Homologous genes are shown in the same color.
(PDF)

S5 Fig Intracellular growth of *L*. *monocytogenes* ST121 strains in human cell lines. Intracellular growth coefficient (IGC) of three human ST121 isolates (L58-55, N12-0367, N13-0119; all harboring a truncated inlA), the food isolate 4423 (truncated inlA), and strain P02-001 (food isolate, full length inlA) and the reference strains EGDe and ScottA in Caco2 (A) and HepG2 cell lines (B). Mean values and standard deviations of the three independent biological replicates are presented. Different letters indicate significant differences (P< 0.05) between the IGC of the strains.
(PDF)

S1 Table Information on strains used in this study. (XLSX)

S2 Table Genetic features of ST121 strains. (XLSX)

S3 Table Average nucleotide identity and tetranucleotide correlation data of ST121 strains. (XLSX)
