# Supplementary material for: Comparative genomics of human and non-human Listeria monocytogenes sequence type 121 strains
Source: PLoS One. 2017 May 4;12(5):e0176857. doi: 10.1371/journal.pone.0176857 (PMC5417603; doi:10.1371/journal.pone.0176857)
Supplement: S1 Fig — Amino acid alignment of full-length and truncated Lmo0435 homologues in representative ST121 L. monocytogenes (AB27, full-length and 4423, truncated) compared to Lmo0435 from EGDe. The predicted signal peptides are highlighted in blue. For 4423, both predicted truncated Lmo0435 homologues are shown. (PDF) [file pone.0176857.s001.pdf]

Figure S1: Amino acid alignment of full-length and truncated Lmo0435 homologues in representative ST121 *L. monocytogenes* (AB27, full-length and 4423, truncated) compared to Lmo0435 from EGDe. The predicted signal peptides are highlighted in blue. For 4423, both predicted truncated Lmo0435 homologues are shown.

```
Lmo0435_1_4423     1 MIRKIFIGILSFTILIQTVIVSNFNVVYASTEEHKQEAIKLEKKSTNNNIICIKGAVKDK
Lmo0435_AB27       1 MIRKIFIGILSFTILIQTVIVSNFNVVYASTEEHKQEAIKLEKKSTNNNIICIKGAVKDK
Lmo0435_EGDe       1 MIRKIFIGILSFTILIQTVIVSNFNVVYATTEEHKQEAIKLEKKSTNNNIICIKGAVKDK
Lmo0435_2_4423     1 ------------------------------------------------------------

Lmo0435_1_4423       ------------------------------------------------------------
Lmo0435_AB27      61 QYKLSLPKSFNLDEKKTGKEVEYNKEKNELTIIGTGEEITLYLLASQTGTYELELKEGDK
Lmo0435_EGDe      61 EYKLSLPKSFNLDEKKTGKEVEYNKEKNELTIIGTGEEITLYLLASQTGKYELELKEGDK
Lmo0435_2_4423     1 ------------------------------------------------------------

Lmo0435_1_4423       ------------------------------------------------------------
Lmo0435_AB27     121 VQAKLDLIVNNVDEEVAKTAEKSNRQLLRSSISDKLFLQADSSKATLANYTEQITFNYSI
Lmo0435_EGDe     121 VQAKLDLIIKNVDEEVAKTVEKSNRQLLRSSISDKLFLQADSSKATLANYTEQITFNYSI
Lmo0435_2_4423     1 ------------------------------------------------------------

Lmo0435_1_4423       ------------------------------------------------------------
Lmo0435_AB27     181 NFLDGSTNLKNGKLVIDFNNSNLELVNYPKDTAANRNIKSTSYSALTGKLTINLVDNISS
Lmo0435_EGDe     181 NFLDGSTNLKNGKLVIDFNNSNLELVNYPKDTAANRNIKLTSYSALTGKLTINLVDNISS
Lmo0435_2_4423     1 --------MKNGKLVIDFNNSNLELVNYPKDTAANRNIKSTSYSALTGKLTINLVDNISS

Lmo0435_1_4423       ------------------------------------------------------------
Lmo0435_AB27     241 GAPFDIPIVVRAGYGAKPGVPMNLKATLSGENSSGGTFTPAEKTTTVNLEENSTNQDYSP
Lmo0435_EGDe     241 GAPFDIPIVVRAGYGAKPGIPMNLKATLSGENSSGGTYTPSEKTTTVNLEESSSNQDYSP
Lmo0435_2_4423    53 GAPFDIPIVVRAGYGAKPGVPMNLKATLSGENSSGGTFTPAEKTTTVNLEENSTNQDYSP

Lmo0435_1_4423       ------------------------------------------------------------
Lmo0435_AB27     301 ITAGDNSWAFNFKEMSYSLKPGGYTIQWPELQKNSLENKSFKNLKLEYLKENGGDVISVN
Lmo0435_EGDe     301 ITAGDNSWAFNLKELSYSLKPGGYTIQWPEIQKKSLENKSFKNLKLEYLKENGGDVISVN
Lmo0435_2_4423   113 ITAGDNSWAFNFKEMSYSLKPGGYTIQWPELQKNSLENKSFKNLKLEYLKENGGDVISVN

Lmo0435_1_4423       ------------------------------------------------------------
Lmo0435_AB27     361 TADPYVVRFGEPYWSQLSTVNGKANVLVNDDEKQVVEYGPINANIYKKIQVSMGAKVPAD
Lmo0435_EGDe     361 TADPYVIRFGEPFWSQLSTVNGKANVLVNDDEKQVVEYGPINANIYQKIQVSMAAKIPAD
Lmo0435_2_4423   173 TADPYVVRFGEPYWSQLSTVNGKANVLVNDDEKQVVEYGPINANIYKKIQVSMGAKVPAD

Lmo0435_1_4423       ------------------------------------------------------------
Lmo0435_AB27     421 AVKGTVYTGTVNVYDGDVFVTSIKIKAEVADSATSIAVDSKVSKTSISEGDIFEWGFMPR
Lmo0435_EGDe     421 GVEGTEYTGTVNVYDEDVFITSIKIKAEVADSATSIAVDSKVSKTSISEGDIFEWGFMPR
Lmo0435_2_4423   233 AVKGTVYTGTVNVYDGDVFVTSIKIKAEVADSATSIAVDSKVSKTSISEGDIFEWGFMPR

Lmo0435_1_4423       ------------------------------------------------------------
Lmo0435_AB27     481 VSSAAPGVNDLEIVAPIPEGIKALSYIPNNNSMASMKKLEYYQNGKWVSMAPQTSSGWDF
Lmo0435_EGDe     481 VSSAAPGVNDLEIVAPIPEGIKALSYIPNNNSMASIKKLEYYQNGKWVSMAPQTSSGWDF
Lmo0435_2_4423   293 VSSAAPGVNDLEIVAPIPEGIKALSYIPNNNSMASMKKLEYYQNGKWVSMAPQTSSGWDF

Lmo0435_1_4423       ------------------------------------------------------------
Lmo0435_AB27     541 SKIDQSVNRIEKLKLTSRDGIINDKDMPPYTHGTIRMQNIGVKAGETFTLRPESITYTDT
Lmo0435_EGDe     541 SKIDQSVNRIEKLKLTSRDGIINDKDMPPYTHGTIRMQNIGVKAGESFTLRPESITYTDP
Lmo0435_2_4423   353 SKIDQSVNRIEKLKLTSRDGIINDKDMPPYTHGTIRMQNIGVKAGETFTLRPESITYTDT

Lmo0435_1_4423       ------------------------------------------------------------
Lmo0435_AB27     601 DKTSKTIDTTTNSYGKNVQVVEKTSAPAKINGEVFLSSTAGTSGKGFESTIFFNGDKIAQ
Lmo0435_EGDe     601 DKTSKTIDTTTNSYGKNVQVVEKTSAPAKINGEVFLSSTAGTSGKGFGSTIFFNGDKIAQ
Lmo0435_2_4423   413 DKTSKTIDTTTNSYGKNVQVVEKTSAPAKINGEVFLSSTAGTSGKGFESTIFFNGDKIAQ

Lmo0435_1_4423       ------------------------------------------------------------
Lmo0435_AB27     661 SVRLGSYGSKLENPYIFVVVPKGIDVETMRNFIQQPYRSTLNYTYAPANGTNTLYPKSSA
Lmo0435_EGDe     661 SVRLGSYGSKLENPYIFVVVPKGIDLETMRNFIQQPYRSTLNYTYAPANGTNTLYPKSSA
Lmo0435_2_4423   473 SVRLGSYGSKLENPYIFVVVPKGIDVETMRNFIQQPYRSTLNYTYAPANGTNTLYPKSSA
```

```
Lmo0435_1_4423         ------------------------------------------------------------
Lmo0435_AB27      721  DVKGKETLSDGSTLYYWEAPDTGLAPGMENCEMLALDLVFKLNKANSGANKVEFGMGSMT
Lmo0435_EGDe      721  DVKGKETLSDGSTLYYWEAPDTGLAPGMENCEMLALDLVLKLNKANSGANKVEFGMGSMT
Lmo0435_2_4423    533  DVKGKETLSDGSTLYYWEAPDTGLAPGMENCEMLALDLVFKLNKANSGANKVEFGMGSMT

Lmo0435_1_4423         ------------------------------------------------------------
Lmo0435_AB27      781  DSSWNLYGAANSGLETKELSNELQSKLPGVTSTKYLSTTRTANIGVSNSIATKMKIKGSQ
Lmo0435_EGDe      781  DSSWNLNGAANSGLETKELSSELQSKLPGVTSTKYLSTTRTANIGVSNSIATKMKIKGSQ
Lmo0435_2_4423    593  DSSWNLYGAANSGLETKELSNELQSKLPGVTSTKYLSTTRTANIGVSNSIATKMKIKGSQ

Lmo0435_1_4423         ------------------------------------------------------------
Lmo0435_AB27      841  DSDFVDVSAKTATTIPGKRVDYNLTFENNGTRSMKNLEIIDILPHKDDQYVLGTGPRGSK
Lmo0435_EGDe      841  DNDFVDVSANTGTTIPGKRVDYNLTFENNGTRSMKNLEIIDILPHKDDQYVLGTGPRGSK
Lmo0435_2_4423    653  DSDFVDVSAKTATTIPGKRVDYNLTFENNGTRSMKNLEIIDILPHKDDQYVLGTGPRGSK

Lmo0435_1_4423         ------------------------------------------------------------
Lmo0435_AB27      901  FSVIPTSEIEVLVNGKKSDSATLEYSTSYTPERFDTNGEDVAGDSWQIVAPTNMADVKSF
Lmo0435_EGDe      901  FSVIPTSEIEVLVNGKKSDSATLEYSTSYTPERFDTNGEDVAGDSWQIVAPTNMADVKSF
Lmo0435_2_4423    713  FSVIPTSEIEVLVNGKKSDSATLEYSTSYTPERFDTNGEDVAGDSWQIVAPTNMADVKSF

Lmo0435_1_4423         ------------------------------------------------------------
Lmo0435_AB27      961  RIKLPNTEFKVGDEITLSFEAIVPTDAPRNDEIAYNSIAYRIDKETGSGTSKLASEPPRG
Lmo0435_EGDe      961  RIKLPNTEFKVGDEITLNFEAIVPTDAPRNDEIAYNSIAYRIDKETGSGTSKLASEPPRG
Lmo0435_2_4423    773  RIKLPNTEFKVGDEITLSFEAIVPTDAPRNDEIAYNSIAYRIDKETGSGTSKLASEPPRG

Lmo0435_1_4423         ------------------------------------------------------------
Lmo0435_AB27     1021  GVKSTNPSTDLNIAGYSFSDLNKNGVKDADELGLNAVKLDLYKKNNNEFEKVETVYTSSD
Lmo0435_EGDe     1021  GVKSTNPSTDLNIAGNSFSDLNKNGVKDADELGLNAVKLDLYKKNNNEFEKVETVYTSSD
Lmo0435_2_4423    833  GVKSTNPSTDLNIAGYSFSDLNKNGVKDADELGLNAVKLDLYKKNNNEFEKVETVYTSSD

Lmo0435_1_4423         ------------------------------------------------------------
Lmo0435_AB27     1081  ALDKEKGLFDFNGLNNGTYKIAAHLPNKNADFITTGPNKIVKDSKDDSIGWITVNNSTEF
Lmo0435_EGDe     1081  ALDKEKGLFDFNGLNNGTYKIAAHLPNKNADFITTGPNKIVKDSKDDSIGWITVNNSTEF
Lmo0435_2_4423    893  ALDKEKGLFDFNGLNNGTYKIAAHLPNKNADFITTGPNKIVKDSKDDSIGWITVNNSTEF

Lmo0435_1_4423         ------------------------------------------------------------
Lmo0435_AB27     1141  TIDDLANGNPKNLVGIQIPIYMATPIKGTVVFVNKDGEPKIASYGQDYEIALFDKDGKEV
Lmo0435_EGDe     1141  TIDDLANGNPKNLVGIQIPIYMATPIKGAVVFVNKDGEPKITSYGQDYEIALFDKDGKEV
Lmo0435_2_4423    953  TIDDLANGNPKNLVGIQIPIYMATPIKGTVVFVNKDGEPKIASYGQDYEIALFDKDGKEV

Lmo0435_1_4423         ------------------------------------------------------------
Lmo0435_AB27     1201  QSAIKTNSKGEFSFNDVAIKNPADFKLKVTAPTGTNFVYSAKNPLFNMSTKEYTLNSVVP
Lmo0435_EGDe     1201  QSAIKTNSKGEFSFNDVAIKNPADFKLKVTAPTGTNFVYSAKNPLFNMSTKEYTLNSVVP
Lmo0435_2_4423   1013  QSAIKTNSKGEFSFNDVAIKNPADFKLKVTAPTGTNFVYSAKNPLFNMSTKEYTLNSVVP

Lmo0435_1_4423         ------------------------------------------------------------
Lmo0435_AB27     1261  GVGGVAEIYITETSKPTTKIILDKAVTPNAITIESSDEATEVTNEWTLEDSDGTVVYTGT
Lmo0435_EGDe     1261  GVGGVAEIYITETSKPTTKIILDKAVTPNAITIESSDEATEVTNEWTLEDSDGTVVYTGT
Lmo0435_2_4423   1073  GVGGVAEIYITETSKPTTKIILDKAVTPNAITIESSDEATEVTNEWTLEDSDGTVVYTGT

Lmo0435_1_4423         ------------------------------------------------------------
Lmo0435_AB27     1321  GNTIRIPNDEGTYIAKNTATDEAGNTASDEKTFDIDYTVPTLTVNQDASAEVNSTEANIG
Lmo0435_EGDe     1321  GNTIRIPNDEGTYIAKNTATDEAGNTASDEKTFDIDYTVPTLTVNQDASAEVNSTEANIG
Lmo0435_2_4423   1133  GNTIRIPNDEGTYIAKNTATDEAGNTASDEKTFDIDYTVPTLTVNQDASAEVNSTEANIG

Lmo0435_1_4423         ------------------------------------------------------------
Lmo0435_AB27     1381  WIKPLNVAATDTHDGNITPVVDYSKVKWDVLGTYPVTVTATDASGNKATQTVNLRIVDTT
Lmo0435_EGDe     1381  WIKPLNVAATDTHDGNITPVVDYSKVKWDVLGTYPVTVTATDASGNKATQTVNLRIVDTT
Lmo0435_2_4423   1193  WIKPLNVAATDTHDGNITPVVDYSKVKWDVLGTYPVTVTATDASGNKATQTVNLRIVDTT
```

```
Lmo0435_1_4423       ------------------------------------------------------------
Lmo0435_AB27    1441 SPTILITNNPLTYSIENMRKLTEQELYTAAGLIGGDNYDLAPGQAVQPNKQPMVFTSNFS
Lmo0435_EGDe    1441 SPTILITNNPLTYSIENMRKLTEQELYTAAGLIGGDNYDLAPGQAVQPNKQPMVFTSNFS
Lmo0435_2_4423  1253 SPTILITNNPLTYSIENMRKLTEQELYTAAGLIGGDNYDLAPGQAVQPNKQPMVFTSNFS

Lmo0435_1_4423       ------------------------------------------------------------
Lmo0435_AB27    1501 TIFGDIASVKAGQYQVQVNLADSSGNQAIPQTITINVVDTVGPVIKADNVSYHVNTTKTE
Lmo0435_EGDe    1501 TIFGDIASVKAGQYQVQVNLADSSGNQAIPQTITINVVDTVGPVIKADNVSYHVNTTKTE
Lmo0435_2_4423  1313 TIFGDIASVKAGQYQVQVNLADSSGNQAIPQTITINVVDTVGPVIKADNVSYHVNTTKTE

Lmo0435_1_4423       ------------------------------------------------------------
Lmo0435_AB27    1561 AEFFQDARLDVTDNNDDTTDLIITSNFAEKVNLNKPGKYEVTITATDTKGNQTTKEITVQ
Lmo0435_EGDe    1561 AEFFQDARLDVTDNNDDTTDLIITSNFAEKVNLNKPGKYEVTITATDTKGNQTTKEITVQ
Lmo0435_2_4423  1373 AEFFQDARLDVTDNNDDTTDLIITSNFAEKVNLNKPGKYEVTITATDTKGNQTTKEITVQ

Lmo0435_1_4423       ------------------------------------------------------------
Lmo0435_AB27    1621 VSKDKPVITADPKISYQGKTEVTEANFLSGVHTEVTDELDGDVKITSDFAEKVDFNKVGT
Lmo0435_EGDe    1621 VSKDKPVITADPKISYQGKIEVTEANFLSGVHAEVTDELDGDVKITSDFAEKVDFNKVGT
Lmo0435_2_4423  1433 VSKDKPVITADPKISYQGKTEVTEANFLSGVHTEVTDELDGDVKITSDFAEKVDFNKVGT

Lmo0435_1_4423       ------------------------------------------------------------
Lmo0435_AB27    1681 YTVTLNAKDEYGNTAEPVRVSVNIFNKIAPTFNNADNKTIEAVNELPSLESIFKIEAKDY
Lmo0435_EGDe    1681 YTVTLNAKDEYGNTAEPVKVSVSIFNKIAPTFNNADNKTIEAVNELPSLESIFKIEAKDY
Lmo0435_2_4423  1493 YTVTLNAKDEYGNTAEPVRVSVNIFNKIAPTFNNADNKTIEAVNELPSLESIFKIEAKDY

Lmo0435_1_4423       ------------------------------------------------------------
Lmo0435_AB27    1741 LSGNKLKVTYTPEQTIKGNVPGEYSIKVTTKDDSGNTAETTVTLTIKDTTGPSIKIAKST
Lmo0435_EGDe    1741 LSGNKLKVTYTPEQTIKGNVPGEYSIKVTTKDDSGNIAETTVTLTIKDTTGPSIKMTKST
Lmo0435_2_4423  1553 LSGNKLKVTYTPEQTIKGNVPGEYSIKVTTKDDSGNTAETTVTLTIKDTTGPSIKIAKST

Lmo0435_1_4423       ------------------------------------------------------------
Lmo0435_AB27    1801 NKLEVQSKAPNWITFFGIKATDIVDGDVTKNIKVDSSEVNLNKVGTYPIYFTVTDALGNE
Lmo0435_EGDe    1801 KKLEVQSKEPNWITFFGIKATDIVDGDVTKNIKVDSSEVNLNKVGTYPIYFTVTDALGNE
Lmo0435_2_4423  1613 NKLEVQSKAPNWITFFGIKATDIVDGDVTKNIKVDSSEVNLNKVGTYPIYFTVTDALGNE

Lmo0435_1_4423       ------------------------------------------------------------
Lmo0435_AB27    1861 SSKLKSTVQIVDTSSPILTIDKVEISYPKGKTVSDKQFLQDIGTKVTNSYGTVKVTTNLS
Lmo0435_EGDe    1861 SSKLKSTVQIVDTSSPELTIDKLEISYPTGKTISDKQFLQDIGTKVTNSYGTVKVTTNLS
Lmo0435_2_4423  1673 SSKLKSTVQIVDTSSPILTIDKVEISYPKGKTVSDKQFLQDIGTKVTNSYGTVKVTTNLS

Lmo0435_1_4423       ------------------------------------------------------------
Lmo0435_AB27    1921 KIVDWDKAGQYKVTVTATNSSGGVAEKTILLTVKNTDSSFIAVPSKDDNKNKPAKNIPKT
Lmo0435_EGDe    1921 KIVDWNKAGKYKVTVTATNSSGGVAEKTILLTVKNSDSSFIAVPSKDDNKNKPAKNIPKT
Lmo0435_2_4423  1733 KIVDWDKAGQYKVTVTATNSSGGVAEKTILLTVKNTDSSFIAVPSKDDNKNKPAKNIPKT

Lmo0435_1_4423       ---------------------------------
Lmo0435_AB27    1981 GDTLNTELIVMGMMLLLVGGWMFLRRKTKVKTK
Lmo0435_EGDe    1981 GDTLNTELIVMGMMLLLVGGWMFLRRKTKVKTK
Lmo0435_2_4423  1793 GDTLNTELIVMGMMLLLVGGWMFLRRKTKVKTK
```
