# Supplementary material for: Comparative genomics of human and non-human Listeria monocytogenes sequence type 121 strains
Source: PLoS One. 2017 May 4;12(5):e0176857. doi: 10.1371/journal.pone.0176857 (PMC5417603; doi:10.1371/journal.pone.0176857)

Figure S2: Alignment of nine ST121 plasmids.
The plasmids were aligned using Mauve. Homologous regions are shown in the same color. The height of the similarity profile within each block corresponds to the average level of conservation in that region of the plasmids.

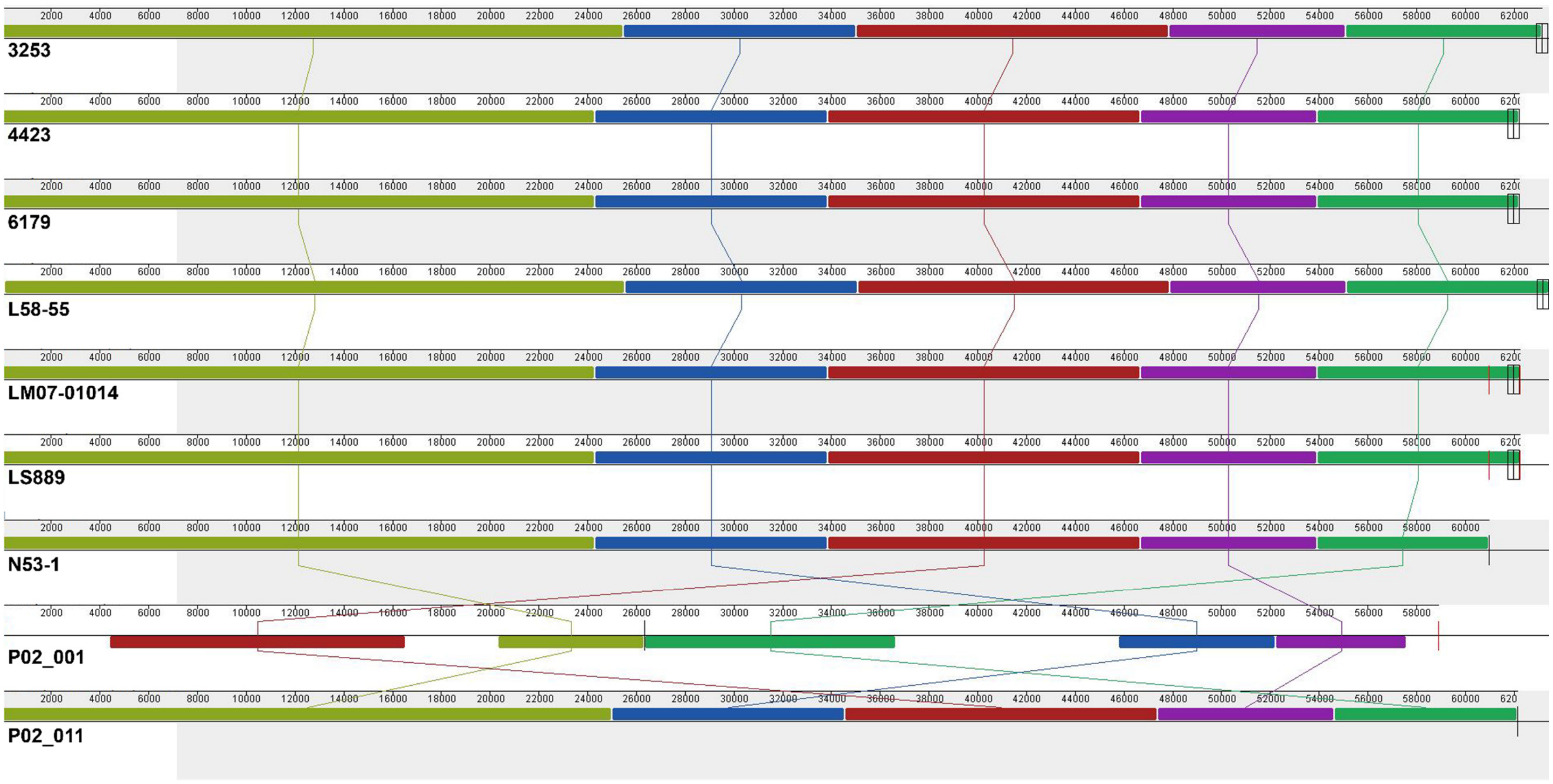

Supplement: S2 Fig — The plasmids were aligned using Mauve. Homologous regions are shown in the same color. The height of the similarity profile within each block corresponds to the average level of conservation in that region of the plasmids. (PDF) [file pone.0176857.s002.pdf]
