# Supplementary material for: Comparative genomics of human and non-human Listeria monocytogenes sequence type 121 strains
Source: PLoS One. 2017 May 4;12(5):e0176857. doi: 10.1371/journal.pone.0176857 (PMC5417603; doi:10.1371/journal.pone.0176857)
Supplement: S3 Fig — Amino acid alignment of selected virulence factors in L. monocytogenes strains 4423 (ST121), EGDe, 10403S, ScottA, F2365. Internalin A (A), Internalin C2 (B), Internalin D (C), Internalin J (D), ActA (E), Lmo0514 (F), Lmo2396 (G), P60/Iap (H), LntA (I). The respective amino acid sequences of strain 4423 are shown as a representative for all other ST121 sequences except for the Lmo0514 (F), where also the Lmo0514 sequence from strain AB27 (a full-length sequence) is shown. The predicted C-terminal LPXTG cell wall anchoring domain in Lmo0514 is highlighted in blue (F). The key amino acids forming the binding motifs of LntA are highlighted in red (I). The ST121 sequences are highlighted in bold. (PDF) [file pone.0176857.s003.pdf]

Figure S3: Amino acid alignment of selected virulence factors in *L. monocytogenes* strains 4423 (ST121), EGDe, 10403S, ScottA, F2365. Internalin A (**A**), Internalin C2 (**B**), Internalin D (**C**), Internalin J (**D**), ActA (**E**), Lmo0514 (**F**), Lmo2396 (**G**), P60/Iap (**H**), LntA (**I**). The respective amino acid sequences of strain 4423 are shown as a representative for all other ST121 sequences except for the Lmo0514 (**F**), where also the Lmo0514 sequence from strain AB27 (a full-length sequence) is shown. The predicted C-terminal LPXTG cell wall anchoring domain in Lmo0514 is highlighted in blue (**F**). Thr key amino acids forming the binding motifs of LntA are highlighted in red (**I**). The ST121 sequences are highlighted in bold.

(A) Internalin A

```
InlA_4423     1 MRKKRYVWLKSILVAILVFGSGVWINTSNGTNAQAATITQDTPINQIFTDAALAEKMKTVLGKTNVTDTV
InlA_EGDe     1 MRKKRYVWLKSILVAILVFGSGVWINTSNGTNAQAATITQDTPINQIFTDTALAEKMKTVLGKTNVTDTV
InlA_10403S   1 MRKKRYVWLKSILVAILVFGSGVWINTSNGTNAQAATITQDTPINQIFTDAALAEKMKTVLGKTNVTDTV
InlA_F2365    1 MRRKRYVWLKSILVAILVFGSGVWINTSNGTNAQAATITQDTPINQIFTDTALAEKMKTVLGKTNVTDTV
InlA_ScottA   1 MRRKRYVWLKSILVAILVFGSGVWINTSNGTNAQAATITQDTPINQIFTDTALAEKMKTVLGKTNVTDTV

InlA_4423    71 SQTDLDQVTTLQADRLGIKSIDGVEYLNNLTQINFSNNQLTDITPLKNLTKLVDILMNNNQIADITPLAN
InlA_EGDe    71 SQTDLDQVTTLQADRLGIKSIDGVEYLNNLTQINFSNNQLTDITPLKNLTKLVDILMNNNQIADITPLAN
InlA_10403S  71 SQTDLDQVTTLQADRLGIKSIDGLEYLNNLTQINFSNNQLTDITPLKDLTKLVDILMNNNQIADITPLAN
InlA_F2365   71 SQTDLDQVTTLQADRLGIKSIDGLEYLNNLTQINFSNNQLTDITPLKDLTKLVDILMNNNQIADITPLAN
InlA_ScottA  71 SQTDLDQVTTLQADRLGIKSIDGLEYLNNLTQINFSNNQLTDITPLKDLTKLVDILMNNNQIADITPLAN

InlA_4423   141 LTNLTGLTLFNNQITDLDPLKNLTNLNRLELSSNTISDISALSGLTSLQQLSFGNQVTDLKPLANLTTLE
InlA_EGDe   141 LTNLTGLTLFNNQITDIDPLKNLTNLNRLELSSNTISDISALSGLTSLQQLSFGNQVTDLKPLANLTTLE
InlA_10403S 141 LTNLTGLTLFNNQITDIDPLKNLTNLNRLELSSNTISDISALSGLTNLQQLSFGNQVTDLKPLANLTTLE
InlA_F2365  141 LSNLTGLTLFNNQITDIDPLKNLTNLNRLELSSNTISDISALSGLTSLQQLSFGNQVTDLKPLANLTTLE
InlA_ScottA 141 LSNLTGLTLFNNQITDIDPLKNLTNLNRLELSSNTISDISALSGLTSLQQLSFGNQVTDLKPLANLTTLE

InlA_4423   211 RLDISSNKVSDISVLAKLTNLESLIATNNQISDITPLGILTNLDELSLNGNQLKDIGTLASLTNLTDLDL
InlA_EGDe   211 RLDISSNKVSDISVLAKLTNLESLIATNNQISDITPLGILTNLDELSLNGNQLKDIGTLASLTNLTDLDL
InlA_10403S 211 RLDISSNKVSDISVLAKLTNLESLIATNNQISDITPLGILTNLDELSLNGNQLKDIGTLASLTNLTDLDL
InlA_F2365  211 RLDISSNKVSDISVLAKLTNLESLIATNNQISDITPLGILTNLDELSLNGNQLKDIGTLASLTNLTDLDL
InlA_ScottA 211 RLDISSNKVSDISVLAKLTNLESLIATNNQISDITPLGILTNLDELSLNGNQLKDIGTLASLTNLTDLDL

InlA_4423   281 ANNQISNLAPLSGLTKLTELKLGANQISNISPLAGLTALTNLELNENQLEDISPISNLKNLTYLTLYFNN
InlA_EGDe   281 ANNQISNLAPLSGLTKLTELKLGANQISNISPLAGLTALTNLELNENQLEDISPISNLKNLTYLTLYFNN
InlA_10403S 281 ANNQISNLAPLSGLTKLTELKLGANQISNISPLAGLTALTNLELNENQLEDISPISNLKNLTYLTLYFNN
InlA_F2365  281 ANNQISNLAPLSGLTKLTELKLGANQISNISPLAGLTALTNLELNENQLEDISPISNLKNLTYLTLYFNN
InlA_ScottA 281 ANNQISNLAPLSGLTKLTELKLGANQISNISPLAGLTALTNLELNENQLEDISPISNLKNLTYLTLYFNN

InlA_4423   351 ISDISPVSSLTKLQRLFFYNNKVSDVSSLANLTNINWLSAGHNQISDLTPLANLTRITQLGLNDQAWTNA
InlA_EGDe   351 ISDISPVSSLTKLQRLFFYNNKVSDVSSLANLTNINWLSAGHNQISDLTPLANLTRITQLGLNDQAWTNA
InlA_10403S 351 ISDISPVSSLTKLQRLFFYNNKVSDVSSLANLTNINWLSAGHNQISDLTPLANLTRITQLGLNDQAWTNA
InlA_F2365  351 ISDISPVSSLTKLQRLFFYNNKVSDVSSLANLTNINWLSAGHNQISDLTPLANLTRITQLGLNDQEWTNP
InlA_ScottA 351 ISDISPVSSLTKLQRLFFYNNKVSDVSSLANLTNINWLSAGHNQISDLTPLANLTRITQLGLNDQEWTNP

InlA_4423   421 PVNYKANVSIPNTVKNVTGALIAPATISDGGSYAEPDITWNLPSYTNEVSYTFNQSVTIGKGTTTFSGTV
InlA_EGDe   421 PVNYKANVSIPNTVKNVTGALIAPATISDGGSYTEPDITWNLPSYTNEVSYTFSQPVTIGKGTTTFSGTV
InlA_10403S 421 PVNYKANVSIPNTVKNVTGALIAPATISDGGSYAEPDITWNLPSYTNEVSYTFSQPVTIGKGTTTFSGTV
InlA_F2365  421 PVNYKVNVSIPNTVKNVTGALIAPATISDGGSYAEPDITWNLPSYTNEVSYTFNQSVTIGKGTTTFSGTV
InlA_ScottA 421 PVNYKVNVSIPNTVKNVTGALIAPATISDGGSYAEPDITWNLPSYTNEVSYTFNQSVTIGKGTTTFSGTV

InlA_4423   491 T---------------------------------------------------------------------
InlA_EGDe   491 TQPLKAIFNVKFHVDGKETTKEVEAGNLLTEPAKPVKEGHTFVGWFDAQTGGTKWNFSTDKMPTNDINLY
InlA_10403S 491 TQPLKAIFNAKFHVDGKETTKEVEAGNLLTEPAKPVKEGHTFVGWFDAQTGGTKWNFSTDKMPTNDINLY
InlA_F2365  491 TQPLKAIFNAKFHVDGKETTKEVEAGNLLTEPAKPVKEGYTFVGWFDAQTGGTKWNFSTDKMPTNDIDLY
InlA_ScottA 491 TQPLKAIFNAKFHVDGKETTKEVEAGNLLTEPAKPVKEGYTFVGWFDAQTGGTKWNFSTDKMPTNDIDLY
```

```
InlA_4423           ----------------------------------------------------------------------
InlA_EGDe      561  AQFSINSYTATFDNDGVTTSQTVDYQGLLQEPTAPTKEGYTFKGWYDAKTGGDKWDFATSKMPAKNITLY
InlA_10403S    561  AQFSINSYTATFENDGVTTSQTVDYQGLLQEPTPPTKEGYTFKGWYDAKTGGDKWDFATSKMPAKNITLY
InlA_F2365     561  AQFSINSYTATFDNDGVTTSQTVDYQGLLQEPTAPTKEGYTFKGWYDAKTGGDKWDFATSKMPAKNITLY
InlA_ScottA    561  AQFSINSYTATFDNDGVTTSQTVDYQGLLQEPTAPTKEGYTFKGWYDAKTGGDKWDFATSKMPAKNITLY

InlA_4423           ----------------------------------------------------------------------
InlA_EGDe      631  AQYSANSYTATFDVDGKSTTQAVDYQGLLKEPKAPTKAGYTFKGWYDEKTDGKKWDFATDKMPANDITLY
InlA_10403S    631  AQYSANSYTATFDVDGKSTTQAVDYQGLLKEPKAPTKAGYTFKGWYDEKTDGKKWDFATDKMPANDITLY
InlA_F2365     631  AQYSANSYTATFDVDGKTTTQAVDYQGLLKEPKTPTKAGYTFKGWYDEKTDGKKWDFATDKMPANDITLY
InlA_ScottA    631  AQYSANSYTATFDVDGKTTTQAVDYQGLLKEPKTPTKAGYTFKGWYDEKTDGKKWDFATDKMPANDITLY

InlA_4423           ----------------------------------------------------------------------
InlA_EGDe      701  AQFTKNPVAPPTTGGNTPPTTNNGGNTTPPSANIPGSDTSNTSTGNSASTTSTMNAYDPYNSKEASLPTT
InlA_10403S    701  AQFTKNPVAPPTTGGNTPPTTNNGGNTTPPSANIPGSDTSNTSTGNSASTTSTMNAYDPYNSKEASLPTT
InlA_F2365     701  AQFTKNPVAPPTTGGNTPPTTNNGGNTTPPSANIPGSNTSNTSTGNSASTTSTMNAYDPYNSKEASLPTT
InlA_ScottA    701  AQFTKNPVAPPTTGGNTPPTTNNGGNTTPPSANIPGSNTSNTSTGNSASTTSTMNAYDPYNSKEASLPTT

InlA_4423           ------------------------------
InlA_EGDe      771  GDSDNALYLLLGLLAVGTAMALTKKARASK
InlA_10403S    771  GDSDNALYLLLGLLAVGTAMALTKKARASK
InlA_F2365     771  GDSDNALYLLIGLLAVGTAVALTKKARASK
InlA_ScottA    771  GDSDNALYLLIGLLAVGTAVALTKKARASK
```

(B) Internalin C2

```
InlC2_4423      1 MKKRWNSVFKLVLMVAAILGISLYVTTSQGAEVRAESIAQPTPINVIFPDPALANAVKTATGKSNVTDTV
inlC2_ScottA    1 MKKRWNSVFKLVLMVAAILGISLYVTTSQGAEVRAESIAQPTPINVIFPDPALANAVKTATGKSNVTDTV
inlC2_F2365     1 MKKRWNSVFKLVLMVAAILGISLYVTTSQGAEVRAESIAQPTPINVIFPDPALANAVKTATGKSNVTDAV
inlC2_10304S    1 MKKRWNSVFKLVLMVTAILGLSLYVTTSQGVEVRAESITQPTAINVIFPDPALANAIKIAAGKSNVTDTV

InlC2_4423     71 TQADLDGIVTLSAFNTGVTTIEGIQYLNNLISLELKDNQITNLTPLKNLTKITELELSGNPLKNVSAIAG
inlC2_ScottA   71 TQADLDGIATLSAFNTGVTTIEGIQYLNNLIGLELKDNQITDLTPLKNLTKITELELSGNPLKNVSAIAG
inlC2_F2365    71 TQADLDGIATLSAFNTGVTTIEGIQYLNNLIGLELKDNQITDLTPLKNLTKITELELSGNPLKNVSAIAG
inlC2_10304S   71 TQADLDGITTLSAFGTGVTTIEGVQYLNNLIGLELKDNQITDLTPLKNLTKITELELSGNPLKNVSAIAG

InlC2_4423    141 LQSIKTLDLTSTQITDVTPLAGLSNLQVLYLDLNQITDISPLAGLTNLQYLSIGNTQVSDLTPLANLSKL
inlC2_ScottA  141 LQSIKTLDLTSTQITDVAPLAGLSNLQVLYLDLNQITDLSPLAGLTNLQYLSIGNNQVNDLTPLANLSKL
inlC2_F2365   141 LQSIKTLDLTSTQITDVTPLAGLSNLQVLYLDLNQITNISPLAGLTNLQYLSIGNNQVNDLTPLANLSKL
inlC2_10304S  141 LQSIKTLDLTSTQITDVTPLAGLSNLQVLYLDLNQITNISPLAGLTNLQYLSIGNAQVSDLTPLANLSKL

InlC2_4423    211 TNLRADDNKISDISPLAGLPNLIEVHLKDNQITDISPLANISNLFIVTLTNQTITNQPVYYQNNLVVPNV
inlC2_ScottA  211 TTLRADDNKISDISPLASLPNLIEVHLKDNQISDVSPLANLSNLFIVTLTNQTITNQPVYYQNNLVVPNV
inlC2_F2365   211 TTLRADDNKISDISPLASLPNLIEVHLKDNQISDVSPLANLSNLFIVTLTNQTITNQPVYYQNNLVVPNV
inlC2_10304S  211 TTLKADDNKISDISPLASLPNLIEVHLKNNQISDVSPLANTSNLFIVTLTNQTITNQPVFYQNNLVVPNI

InlC2_4423    281 VKGPSGAPIAPATISDNGTYASPNLTWNLTSFINNVSYTFNQSVTFKNTTVPFSGTVTQPLTEAYTAVFD
inlC2_ScottA  281 VKGPSGAPIAPATISDNGTYASPNLTWNLTSFINNVSYTFNQSVAFKNTTVPFSGTVTQPLTEAYTAVFD
inlC2_F2365   281 VKGSSGAPIAPATISDNGTYASPNLTWNLTSFINNVSYTFNQSVAFKNTTVPFSGTVTQPLTEAYTAVFD
inlC2_10304S  281 VKGPSGAPIAPATISDNGTYASPNLTWNLTSFINNVSYTFNQSVTFKNTTVPFSGTVTQPLTEAYTAVFD

InlC2_4423    351 VDGKQTSMTVGANELIKEPTAPTKEGYTFTGWYDAKTGGNKWDFSTDKMPAENITLYAQFTINSYTASFD
inlC2_ScottA  351 VDGKQTSVTVGANELIKEPTAPTKEGYTFTGWYDAKTGGNKWDFSTDKMPAENITLYAQFTINSYTATFD
inlC2_F2365   351 VDGKQTSVTVGANELIKEPTAPTKEGYTFTGWYDAKTGGNKWDFSTDKMPAENITLYAQFTINSYTATFD
inlC2_10304S  351 VDGKQTSVTVGANELIKEPTAPTKEGYTFTGWYDAKTGGNKWDFGVDKMPAENITLYAQFTINSYTASFD

InlC2_4423    421 NDGKITTQKVTYQSLLEEPAAPTKDGYTFKGWYDAKTGGTKWDFATGKMPAGNITLYAQFTKNDSPNPND
inlC2_ScottA  421 NDGKITTQKVTYQSLLEEPAAPTKAGYTFKGWYDAKTGGTKWDFATGKMPAGNITLYAQFTKNDSPNPND
inlC2_F2365   421 NDGKITTQKVTYQSLLEEPAAPTKAGYTFKGWYDAKTGGTKWDFATGKMPAGNITLYAQFTKNDSPNPND
inlC2_10304S  421 NDGKLTTQKVTYQSLLEEPAAPTKTGYTFKGWYDAKTGGTKWDFATGKMPAGNITLYAQFTKNDSPNPND

InlC2_4423    491 PTPNTPTGNGDGTSNPSNSGGNTTLPTAGDENTMLPIFIGVFLLGTATLILRKTIKVK
inlC2_ScottA  491 PTPNTPTGNGDGTSNPSDSGGNTTLPTAGDENTMLPIFIGVFLLGTATLILRKTIKVK
inlC2_F2365   491 PTPNTPTGNGDGTSNPSDSGGNTTLPTAGDENTMLPIFIGVFLLGTATLILRKTIKVK
inlC2_10304S  491 PTPNTPTGNGDGTSNPSNSGGNTTLPTAGDENTMLPIFIGVFLLGTATLILRKTIKVK
```

(C) Internalin D

```
InlD_4423       1 MKRNKTALRILVTLAVVMAISFWVGKSSK-EVQAAEIGQPTPINEIFTDENLANAIKITLSKPNTTSDVS
InlD_10403S     1 MKRNKTALRILVTLAVVMAISFWVGTSSK-EVQAAEIGQPTPINEIFTDENLANAIKTTLSKPSTASAVS
InlD_ScottA     1 MKRKKTALRILVTLAVVMAISFWVGTSSKEEVQAAVIDQPTPINEIFTDENLANAIKATLNKPSTTSDVS
InlD_F2365      1 MKRKKTALRILVTLAVVMAISFWVGTSSKEEVQAAVIDQPTPINEIFTDENLANAIKATLNKPSTTSDVS

InlD_4423      70 QTELDSISEVTAESSNIASLEGTQYLNNLDTLVLNNNKITTLNPLAGLTKLSILEASNNQLSDISALSNV
InlD_10403S    70 QVELDSVRDVTAESSNIASLEGVQYLNNLDTLVLNNNKITDLNPLAGLTKLSILEASNNQLSDISALSNV
InlD_ScottA    71 QAELDSISEVTAESSNIASLEGAQYLNNIDTLILNNNKITDLNSLAGLSKLRILEANDNQLSDISALANV
InlD_F2365     71 QAELDSISEVTAESSNIASLEGAQYLNNIDTLILNNNKITDLNSLAGLSKLRILEANDNQLSDISALANV

InlD_4423     140 TNLHQLRLDGNQIKQLNGVSNLINLETIELSNNQITAISPVSGLKNLVGLGIDNNKISDLSPISGLSKLN
InlD_10403S   140 TNLHQLRLDGNQIKQLNGVSNLINLETIELSNNQITAISPVSGLKNLVGLGIDNNKISDLSPISGLSKLN
InlD_ScottA   141 TSLHQLRLAGNQIKQLNGVSNLINLETIELSNNQITDISPVSGLKNLIGLGIDYNDISDLSPIAGLAKLS
InlD_F2365    141 TSLHQLRLAGNQIKQLNGVSNLINLETIELSNNQITDISPVSGLKNLIGLGIDYNDISDLSPIAGLAKLS

InlD_4423     210 HLTADSNQISDLRPLSNLAAMEVMRLDGNQISDVTPIANLANLNYVFLAENQISDISSLQPLFNSPNFFG
InlD_10403S   210 HLTADSNQISDLRPLSNLAAMEVMRLDGNQISDVTPIANLANLNYVFLAENQISDISSLQPLFNSPNFFG
InlD_ScottA   211 HLTADNNQISDLSPISSLGAMEIMRLDKNQISDVTPIANLANLSYVFLAENQISDISSLQPLFNSPNFFG
InlD_F2365    211 HLTADNNQISDLSPISSLGAMEIMRLDKNQISDVTPIANLANLSYVFLAENQISDISSLQPLFNSPNFFG

InlD_4423     280 ITLDNQKITSEPVLYQQELVVPNYIKDEMGALIAPATISDNGVYASPNINWNLPNYTNQVSYTFNKQLAY
InlD_10403S   280 ITLDNQKITSEPVLYQQELVVPNNIKDEMGALIAPATISDNGVYASPNINWNLPNYTNQVSYTFNKQLAY
InlD_ScottA   281 ITLDNQKITSEPVLYQQELVVPNNIKDEMGALISPDTISDNGVYASPNIKWNLANYTNQVSYTFNKQLAQ
InlD_F2365    281 ITLDNQKITSEPVLYQQELVVPNNIKDEMGALISPDTISDNGVYASPNIKWNLANYTNQVSYTFNKQLAQ

InlD_4423     350 GSFSGTVTQPLHNAYTATFDVDGVKTNEAVEETKLLQEPIAPTKEGYTFTGWYDAKTGGTKWDFATDKMP
InlD_10403S   350 GSFSGTVTQPLHNAYTATFDVDGVKTNEAVEETKLLQEPIAPTKEGYTFTGWYDAKTGGNKWDFATDKMP
InlD_ScottA   351 GSFSGTVTQPLHNAYTATFDVDGVKTNEVVEETKLLQEPTAPTKEGYTFTGWYDAKTGGNKWDFATDKMP
InlD_F2365    351 GSFSGTVTQPLHNAYTATFDVDGVKTNEVVEETKLLQEPTAPTKEGYTFTGWYDAKTGGNKWDFATDKMP

InlD_4423     420 AEDITLYAQFTINSYTATFDIDGKLTTQKVTYQSLLEEPVAPTKDGYTFTGWYDAKTGGNKWDFANGKMP
InlD_10403S   420 AEDITLYAQFTINSYTATFDIDGKLTTQKVTYQSLLEEPVAPTKDGYTFTGWYDAKTGGTKWDFATGKMP
InlD_ScottA   421 AEDITLYAQFTINSYTATFDIDGKLTTQKVTYQSLLEEPAAPTKDGYTFIGWYDAKTGGTKWDFATDKMP
InlD_F2365    421 AEDITLYAQFTINSYTATFDIDGKLTTQKVTYQSLLEEPAAPTKDGYTFIGWYDAKTGGTKWDFATDKMP

InlD_4423     490 AGNITLYAQFTKNDSPNPNDPTPNTPTGNGEGTSNPSNSGGNTTLPTAGDENTMLPIFIGVFLLGTATLI
InlD_10403S   490 AGNITLYAQFTKNDNPNPDDPTTNTPTGNGDGTSNPSNSGGNTTLPTAGDENTMLPIFIGVFLLGTATLI
InlD_ScottA   491 AGNITLYAQFTKNATPDSNDPTTVTPTGNGNGTSTPSNSGGNTTLPTAGDENTMLPIFVGVFLLGTATLI
InlD_F2365    491 AGNITLYAQFTKNATPDSNDPTTVTPTGNGNGTSTPSNSGGNTTLPTAGDENTMLPIFVGVFLLGTATLI

InlD_4423     560 LRKTIKVK
InlD_10403S   560 LRKTIKVK
InlD_ScottA   561 LRKTIKVK
InlD_F2365    561 LRKTIKVK
```

(D) Internalin J

```
InlJ_4423     1 MKTTKIVIASLVSLTMVSNPLLTFAATNDVIDNTTEITTDKETSSTQPTIKNTLKAGQTQSFNDWFPDDN
InlJ_F2365    1 MKTSKIIIASLVSLTLVSNPILTFAATNDVIDSTTEITTDKEISSTQPTIKTTLKAGQTQSFNDWFPDDN
InlJ_EGDe     1 MKTTKIVIASLVSLTMVSNPLLTFAATNDVIDNTTEITTDKETSSTQPTIKNTLKAGQTQSFNDWFPDDN

InlJ_4423    71 FASEVAAVFEMQATDTISEEQLATLTSLDCHNSSITDMTGIEKLTGLTKLICTSNNITTLDLSKNTNLTY
InlJ_F2365   71 FASEVAAAFEMQATDTISEEQLATLTSLDCHNSSIADMTGIEKLTGLTKLICTYNNITTLDLSQNTNLTY
InlJ_EGDe    71 FASEVAAAFEMQATDTISEEQLATLTSLDCHNSSITDMTGIEKLTGLTKLICTSNNITTLDLSQNTNLTY

InlJ_4423   141 LECNSNKLTNLDVTPLTKLTYLNCDTNKLTKLDVSQNPLLTYLNCARNTLTEIDVSHNTQLTELDCHLNK
InlJ_F2365  141 LACDSNKLTNLDVTPLTKLTYLNCDTNKLTKIDVSQNPLLTYLNCARNTLTEIDVSHNTQLTELDCHLNK
InlJ_EGDe   141 LACDSNKLTNLDVTPLTKLTYLNCDTNKLTKLDVSQNPLLTYLNCARNTLTEIDVSHNTQLTELDCHLNK

InlJ_4423   211 KITKLDVTPQTQLTTLDCSFNKITELDVSQNKLLNRLNCDTNNLTKLDLNQNIKLTFLDCSSNKLTEIDV
InlJ_F2365  211 KITKLDVTPQTQLTTLDCSFNKITALDVSQNKLLNRLNCDTNNITKLDLNQNIQLTFLNCSSNKLTEIDV
InlJ_EGDe   211 KITKLDVTPQTQLTTLDCSFNKITELDVSQNKLLNRLNCDTNNITKLDLNQNIQLTFLDCSSNKLTEIDV

InlJ_4423   281 TPLTQLTYFDCGVNPLTELDVSTLSKLTTLECIQTDLLEIDLTHNTQLTNFKAEGCRKIKDLDVTHNTQL
InlJ_F2365  281 TPLTQLTYFDCSVNPLTELDVSTLSKLTTLHCIQTDLLEIDLTHNTQLIYFQAEGCRKIKELDVTHNTQL
InlJ_EGDe   281 TPLTQLTYFDCSVNPLTELDVSTLSKLTTLHCIQTDLLEIDLTHNTQLIYFQAEGCRKIKELDVTHNTQL

InlJ_4423   351 YSLDCQGAGITELDLSQNPKLVYLYLNNTELTKLDVSPNTKLKKLFCENTHVQDFSSMRNIAALNNNLYA
InlJ_F2365  351 YLLDCQAAGITELDLSQNPKLVYLYLNNTELTKLDVSHNTKLKSLSCVNAHIQDFSSVGKIPVLNNNLDA
InlJ_EGDe   351 YLLDCQAAGITELDLSQNPKLVYLYLNNTELTELDVSHNTKLKSLSCVNAHIQDFSSVGKIPALNNNFEA

InlJ_4423   421 EGQTITMPKETLINNSLTIAVSPDLLDQFGNPMNIEPGDGGVYDQATNTITWENLSTDNPAVTYTFTSAN
InlJ_F2365  421 EGQTITMPKETLTNNSLTIAVSPDLLDQFGNPMNIEPGDGGVYDQATNTITWENLSTDNPAVTYTFTSEN
InlJ_EGDe   421 EGQTITMPKETLTNNSLTIAVSPDLLDQFGNPMNIEPGDGGVYDQATNTITWENLSTDNPAVTYTFTSEN

InlJ_4423   491 GAIVGTVTTPFEAPQPIKGEDVTVHYLDDKGEKLADDEVLSGNLDDPYTSSAKDIPDYTLTTTPDNATGT
InlJ_F2365  491 GAIVGTVTTPFEAPQPIKGEDVTVHYLDDKGEKLAADEVLSGNLDDPYTSSAKDIPDYTLTTTPDNATGT
InlJ_EGDe   491 GAIVGTVTTPFEAPQPIKGEDVTVHYLDDKGEKLADDEVLSGNLDDPYTSSAKDIPDYTLTTTPDNATGT

InlJ_4423   561 FTTTSQSVTYVYTKNIVAAEPVTVNYVDDTGKTLAPSETLNGNVGDTYNATAKQIDGYTLTTTPNNATGT
InlJ_F2365  561 FTTTSQSVTYVYTKNIVAAEPVTVNYVDDTGKTLAPSETLNGNVGDTYNATAKQIDGYTLSTTPNNATGT
InlJ_EGDe   561 FTTTSQSVTYVYTKNIVAAEPVTVNYVDD-----------------------------------------

InlJ_4423   631 FNTSSQTVTYVYTKNIEAAEPVTVNYVDATGKTLAPSETLNGNVGDTYNATAKQIDGYTLSTEPTNATGQ
InlJ_F2365  631 FNTSSQTVTYVYTKNIVAAEPVTVNYVDDTGKTLAPSETLNGNVGDTYNATAKQIDGYTLSAEPTNATGQ
InlJ_EGDe   590 -----------------------------TGKTLSPSEILNGNVGDTYNATAKQIDGYTLSAEPTNATGQ

InlJ_4423   701 FTSSAQTVNYIYTKNPAPEKGVVEIHYVDENNKQLSSATEISGTVGNNYTTEPKTIDGYTLTTTPDNATG
InlJ_F2365  701 FTSSAQTVNYIYTKNPAPEKGVVEIHYVDENNKQLSSATKISGTVGDNYTTEPKNIDGYTLTTTPDNATG
InlJ_EGDe   631 FTSSAQTVNYIYTKNPAPEKGVVEIHYVDEDNKQLNSTTEISGTIGDNYTTEPKTIEGYTLTTTPGNATG

InlJ_4423   771 TFNTSSQTVTYVYTKNIEAAEPVTVNYVDANGKTLAPSETLNGTIGDTYNATAKQIDGYTLSAEPTNATG
InlJ_F2365  771 TFNTSSQTVTYVYTKNIVAAEPVTVNYVDANGKTLAPSETLNGTIGDTYNATAKQIDGYTLSAEPTNATG
InlJ_EGDe   701 TFTTGSQTVTYVYTKNIEAAEPITVNYVDANGKTLAPSETLNGNVGDTYKATAKQIDGYTLSAEPTNATG

InlJ_4423   841 QFTNSAQTVNYIYTKNTNIDQPLPDKKPTNTTPTKPSNLKTTEVKKASDTLPKTGDSAPWKSALLGVFLS
InlJ_F2365  841 QFTNSAQTVNYIYTKNTNIDQPLPDKK-----TTKPSNLKTTEVKKASDTLPKTGDSTPWKSALLGVFLS
InlJ_EGDe   771 QFTSSAQTVNYIYTKNTNTDQPLPTKKPTNTTPTKPSNLKTTEVKKASDTLPKTGDSAPWKSALLGVFLS

InlJ_4423   911 STALVIWKKKK
InlJ_F2365  906 STALVIWKKKK
InlJ_EGDe   841 STALVIWKKKK
```

(E) ActA

```
ActA_4423       1 MGLNRFMRAMMVVFITANCITINPDIIFAATDSEDSSLNTDEWEEEKTEEQPSEVNTGPRYETAREVSSR
ActA_EGDe       1 MGLNRFMRAMMVVFITANCITINPDIIFAATDSEDSSLNTDEWEEEKTEEQPSEVNTGPRYETAREVSSR
ActA_10403S     1 ------MRAMMVVFITANCITINPDIIFAATDSEDSSLNTDEWEEEKTEEQPSEVNTGPRYETAREVSSR
ActA_F2365      1 MGLNRFMRAMMVVFITANCITINPDIIFAATDSEDSSLNTDEWEEEKTEEQPSEVNTGPRYETAREVSSR
ActA_ScottA     1 ------MRAMMVVFITANCITINPDIIFAATDSEDSSLNTDEWEEEKTEEQPSEVNTGPRYETAREVSSR

ActA_4423      71 DIKELEKSNKVKNTNKADLIAMLKAKAEKGPNINNNNSEQTENAAINEEASGADRPAIQVERRHPGLPSD
ActA_EGDe      71 DIKELEKSNKVRNTNKADLIAMLKEKAEKGPNINNNNSEQTENAAINEEASGADRPAIQVERRHPGLPSD
ActA_10403S    65 DIEELEKSNKVKNTNKADLIAMLKAKAEKGPNNNNNNGEQTGNVAINEEASGVDRPTLQVERRHPGLSSD
ActA_F2365     71 DIEELEKSNKVKNTNKADLIAMLKAKAEKGPNINNNNSEQSDNVAINEEASGSDRPAIQVERRHPGLPSD
ActA_ScottA    65 DIEELEKSNKVKNTNKADLIAMLKAKAEKGPNINNNNSEQSENVAINEEASGSDRPAIQVERRHPGLPSD

ActA_4423     141 SAAEIKKRRKAIASSDSELESLTYPDKPTKVNKKKVAKESVADASESDLDSSMQSADESTPQPLKANQKP
ActA_EGDe     141 SAAEIKKRRKAIASSDSELESLTYPDKPTKVNKKKVAKESVADASESDLDSSMQSADESSPQPLKANQQP
ActA_10403S   135 SAAEIKKRRKAIASSDSELESLTYPDKPTKANKRKVAKESVVDASESDLDSSMQSADESTPQPLKANQKP
ActA_F2365    141 SAAEIKKRRKAIASSDSELESLTYPDKPTKATKKKVAKASVTDTSESDLDSSMQSADESTPQPLKANQQP
ActA_ScottA   135 SAAEIKKRRKAIASSDSELESLTYPDKPTKATKKKVAKASVTDTSESDLDSSMQSADESTPQPLKANQQP

ActA_4423     211 FFPKVFKKIKDAGKWVRDKIDENPEVKKAIVDKSAGLIDQLLTKKKSEEVNASDFPPPPTDEELRLALPE
ActA_EGDe     211 FFPKVFKKIKDAGKWVRDKIDENPEVKKAIVDKSAGLIDQLLTKKKSEEVNASDFPPPPTDEELRLALPE
ActA_10403S   205 FFPKVFKKIKDAGKWVRDKIDENPEVKKAIVDKSAGLIDQLLTKKKSEEVNASDFPPPPTDEELRLALPE
ActA_F2365    211 FFPKVFKKIKDAGKWVRDKIDENPEVKKAIVDKSAGLIDQLLTKKKNEEVNASD----------------
ActA_ScottA   205 FFPKVFKKIKDAGKWVRDKIDENPEVKKAIVDKSAGLIDQLLTKKKNEEVNASD----------------

ActA_4423     281 TPMLLGFNAP-------------------------TP----------SEPSSFEFPPPPTEDELEIMRET
ActA_EGDe     281 TPMLLGFNAPATSEPSSFEFPPPPTDEELRLALPETPMLLGFNAPATSEPSSFEFPPPPTEDELEIIRET
ActA_10403S   275 TPMLLGFNAPTPSEPSSFEFPPPPTDEELRLALPETPMLLGFNAPATSEPSSFEFPPPPTEDELEIMRET
ActA_F2365    265 -------------------FPPPPTDEELRLALPETPMLLGFNAPATSEPSSFEFPPPPTEDELEIMRET
ActA_ScottA   259 -------------------FPPPPTDEELRLALPETPMLLGFNAPATSEPSSFEFPPPPTEDELEIMRET

ActA_4423     316 APSLDSSFTSGDLASLRSAINRHSENFSDFPPIPTEEELNGRGGRPTSEEFSSMNSGDFTDDENSETTEA
ActA_EGDe     351 ASSLDSSFTRGDLASLRNAINRHSQNFSDFPPIPTEEELNGRGGRPTSEEFSSLNSGDFTDDENSETTEE
ActA_10403S   345 APSLDSSFTSGDLASLRSAINRHSENFSDFPPIPTEEELNGRGGRPTSEEFSSLNSGDFTDDENSETTEE
ActA_F2365    316 APSLDSSFTSGDLASLRSAINRHSQNFSDFPLMPTEEELNGRGGIPTSEEFSSLNSGDFTDDENSETTEE
ActA_ScottA   310 APSLDSSFTSGDLASSRSAINRHSQNFSDFPPMPTEEELNGRGGIPTSEEFSSLNSGDFTDDENSETTEE

ActA_4423     386 EIDRLADLRDRGTGKHSRNAGFLPLNPFISSPVPSLTPKVPKISAPALISDITKKAPFKNPSQPLNVFNK
ActA_EGDe     421 EIDRLADLRDRGTGKHSRNAGFLPLNPFASSPVPSLSPKVSKISAPALISDITKKTPFKNPSQPLNVFNK
ActA_10403S   415 EIDRLADLRDRGTGKHSRNAGFLPLNPFISSPVPSLTPKVPKISAPALISDITKKAPFKNPSQPLNVFNK
ActA_F2365    386 EIDRLADLRDRGTGKHSRNAGFLPLNPFTSSPVPSLSPKVSKISAPALITDITKKAPFKNPPQPLNVFNK
ActA_ScottA   380 EIDRLADLRDRGTGKHSRNAGFLPLNPFTSSPVPSLTPKVPKISAPALITDITKKAPFKNPPQPLNVFNK

ActA_4423     456 KTTTKTVTKKPTPVKTAPKLAELPATKPQETVLRENKTPFIEKQAETNKQSINMPSLPVIQKEATESDKE
ActA_EGDe     491 KTTTKTVTKKPTPVKTAPKLAELPATKPQETVLRENKTPFIEKQAETNKQSINMPSLPVIQKEATESDKE
ActA_10403S   485 KTTTKTVTKKPTPVKTAPKLAELPATKPQETVLRENKTPFIEKQAETNKQSINMPSLPVIQKEATESDKE
ActA_F2365    456 KTTTKTAPKKITPVNTAPKLAALPITKAQETALGENKAPFIEKQAETNNRPIDMPSLPVIQKEVTERNKE
ActA_ScottA   450 KTTTKTAPKKITPVNTAPKLAALPITKAQETELGENKAPFIEKQAETNNRPIDMPSLPVIQKEVTERNKE

ActA_4423     526 EMKPQTEEKMVEESESANNANGKNRSAGIEEGKLIAKSAEDEKAKEEPGNHTTLILAMLAIGVFSLGAFI
ActA_EGDe     561 EMKPQTEEKMVEESESANNANGKNRSAGIEEGKLIAKSAEDEKAKEEPGNHTTLILAMLAIGVFSLGAFI
ActA_10403S   555 EMKPQTEEKMVEESESANNANGKNRSAGIEEGKLIAKSAEDEKAKEEPGNHTTLILAMLAIGVFSLGAFI
ActA_F2365    526 EMKPQTEEKVVGESEPANNVNGKKRSAGIEEGKLIAKSAEDEKAKEEPVNHTTLILAMLAIGVFSLGAVI
ActA_ScottA   520 EMKPQTEEKVVGESEPANNVNGKKRSAGIEEGKLIAKSAEDEKAKEEPVNHTTLILAMLAIGVFSLGAVI

ActA_4423     596 KIIQLRKNN
ActA_EGDe     631 KIIQLRKNN
ActA_10403S   625 KIIQLRKNN
ActA_F2365    596 KIIQLRKNS
ActA_ScottA   590 KIIQLRKNS
```

(F) Lmo0514

```
Lmo0514_EGDe      1  MKKALKLFLATICMFIMIYPSTAAHAEETNIVNIPDANLKTYLNGLLKQASDAPITKTQMNTIQTVTLSG
Lmo0514_10403S    1  MKKALKLFLAMICMFIMIYPSTAAHAEETNIVNIPDANLKTYLNGLLKQASDAPITKTQMNTIQTVTLSG
Lmo0514_4423      1  MKKALKLFLAMICMFIMIYPSTAAHAEETNIVNIPDANLKTYLNGLLKQASDAPITKTQMNTIQTVTLSG
Lmo0514_AB27      1  MKKALKLFLAMICMFIMIYPSTAAHAEETNIVNIPDANLKTYLNGLLKQASDAPITKTQMNTIQTVTLSG
Lmo0514_F2365     1  MKKALKLFLATICMFIMIYPSTAAHAEETNIVNIPDANLKTYLNGLLKQASDAPITKTQMNTIQTVTLSG
Lmo0514_ScottA    1  MKKALKLFLATICMFIMIYPSTAAHAEETNIVNIPDANLKTYLNGLLKQASDAPITKTQMNTIQTVTLSG

Lmo0514_EGDe     71  STYTDLTGLEEAGNLVTLSLNNTNIQTLEPIKNQTSLTYLTVGGDNVKDSLFVDLNGLVNLQSLSINGSE
Lmo0514_10403S   71  STYTDLTGLEEAGNLVTLSINNTNIQTLEPIKNQTSLTYLTVGGDNVKDSLFVDLNGLVNLQSLSINGSE
Lmo0514_4423     71  STYTDLTGLEEAGNLVTLSINNTNIQTLEPIKNQTSLTYLTVGGDNVKDSLFVDLNGLVNLQSLSINGSE
Lmo0514_AB27     71  STYTDLTGLEEAGNLVTLSINNTNIQTLEPIKNQTSLTYLTVGGDNVKDSLFVDLNGLVNLQSLSINGSE
Lmo0514_F2365    71  STYTDLTGLEEAGNLVTLSLNNTNIQTLEPIKNQTSLTYLTVGGDNVKDSLFVDLNGLVNLQSLSISGSE
Lmo0514_ScottA   71  STYTDLTGLEEAGNLVTLSLNNTNIQTLEPIKNQTSLTYLTVGGDNVKDSLFVDLNGLVNLQSLSISGSE

Lmo0514_EGDe    141  VTHNVFKTFNKLPKLTYLYAQNSMKITDISALASLPALTTLFLQFDGIDDFRPLNDFESFKNGNLKALAA
Lmo0514_10403S  141  VTHNVFKTFNKLPKLTYLYAQNSMKITDISALASLPALTTLFLQFDGIDDFRPLNDFESFKNGNLKALAA
Lmo0514_4423    141  VTHNVFKTFNKLPKLTYLYAQNSMKITDISALASLPALTTLFLQFDGIDDFRPLNDFESFKNGNLKALAA
Lmo0514_AB27    141  VTHNVFKTFNKLPKLTYLYAQNSMKITDISALASLPALTTLFLQFDGIDDFRPLNDFESFKNGNLKALAA
Lmo0514_F2365   141  VTHNVFKTFNKLPKLTYLYAQNSMKITDISALASLPALTTLFLQFDGIDDFRPLNDFESFKNGNLKALAA
Lmo0514_ScottA  141  VTHNVFKTFNKLPKLTYLYAQNSMKITDISALASLPALTTLFLQFDGIDDFRPLNDFESFKNGNLKALAA

Lmo0514_EGDe    211  FGQNTGRTNPRITLKSGKLDYNETNQTLYLPFSMMPKPLTSFDGTVAPFSKSTSASNTYLGFNDVALPSS
Lmo0514_10403S  211  FGQNTGRTNPRITLKSGKLDYDETNQTLYLPFSMMPKPLTSFDGTVAPFSKSTSASNTYLGFNDVALPSS
Lmo0514_4423    211  FGQNTGRTNPRITLKSGKLDYNETNQTLYLPFSMMPKPLTSFDGTVAPFSKSTSASNTYLGFNDVALPSA
Lmo0514_AB27    211  FGQNTGRTNPRITLKSGKLDYNETNQTLYLPFSMMPKPLTSFDGTVAPFSKSTSASNTYLGFNDVALPSA
Lmo0514_F2365   211  FGQNTGRTNPRITLKSGKLDYNETNQTLYLPFSMMPKPLTSFDGTVAPFSKSTSASNTYLGFNDVALPSA
Lmo0514_ScottA  211  FGQNTGRTNPRITLKSGKLDYNETNQTLYLPFSMMPKPLTSFDGTVAPFSKSTSASNTYLGFNDVALPSA

Lmo0514_EGDe    281  RLSITDDGITVSGVTKEEFDNLDEIEYNARYDFPTGSYPTPPSMTSYTISSGTYDQYFDISHTLDLTADE
Lmo0514_10403S  281  RLSITDDGITVSGVTKEEFDNLDEIEYNARYDFPTGSYPTPPSMTSYTISSGTYDQYFDISHTLDLMADE
Lmo0514_4423    281  RLSITEDGITVSGVTKEEFDNLDEIEYNARYDFPTGSYPTPPSMTSYTISSGTYDQYFDISHTLDLTADE
Lmo0514_AB27    281  RLSITEDGITVSGVTKEEFDNLDEIEYNARYDFPTGSYPTPPSMTSYTISSGTYDQYFDISHTLDLTADE
Lmo0514_F2365   281  RLSITDDGITVSGVTKEEFDNLDEIEYNARYDFPTGSYPTPPSMNSYTISSGTYDQYFDISHTLDLTADE
Lmo0514_ScottA  281  RLSITDDGITVSGVTKEEFDNLDEIEYNARYDFPTGSYPTPPSMNSYTISSGTYDQYFDISHTLDLTADE

Lmo0514_EGDe    351  SFDYNQYDTTSEEQFLKDVHAETDDGTAVKSDFDQVVKLDVPGEYTVTLNAENAAGLKATPVTVKVTVHE
Lmo0514_10403S  351  SFDYNQYDTTSEEQFLKDVHAETDDGTAVKSDFDQVVKLDVPGEYTVTLNAENAAGLKATPVTVKVTVHE
Lmo0514_4423    351  SFDYNQYDTTSEEQFLKDVHAETDDGTAVKSDFDQVVKLDVPGEYTVTLNAENAAGLKATPVTVKVTVHE
Lmo0514_AB27    351  SFDYNQYDTTSEEQFLKDVHAETDDGTAVKSDFDQVVKLDVPGEYTVTLNAENAAGLKATPVTVKVTVHE
Lmo0514_F2365   351  SFDYNQYDATSEEQFLKDVHAETDDGTAVKSDFDQVVKLDVPGEYTVTLNAENAAGLKATPVTVKVTVHE
Lmo0514_ScottA  351  SFDYNQYDATSEEQFLKDVHAETDDGTAVKSDFDQVVKLDVPGEYTVTLNAENAAGLKATPVTVKVTVHE

Lmo0514_EGDe    421  KPVITSDSQISYKKETTKSVDEFLKEIHGSVTGNAVLTSDFDKVVDLNTPGEYTVTLNAINDRGQKADPV
Lmo0514_10403S  421  KPVITSDSQISYKKETTKSVDEFLKEIHGSVTGNAVLTSDFDKVVDLNTPGEYTVTLNAINDRGQKADPV
Lmo0514_4423    421  KPVITSDSQISYKKETTKSVDEFLKEIHGSVTGNAVLTSDFDKVVDLNTPGEYTVTLNAINDRGQKADPV
Lmo0514_AB27    421  KPVITSDSQISYKKETTKSVDEFLKEIHGSVTGNAVLTSDFDKVVDLNTPGEYTVTLNAINDRGQKADPV
Lmo0514_F2365   421  KPVITADSQISYKKESTKSVDEFLQEIHSSVTGNAVLTSDFDKVVDLNTPGEYTVTLNAINDRGQKADPV
Lmo0514_ScottA  421  KPVITADSQISYKKESTKSVDEFLQEIHSSVTGNAVLTSDFDKVVDLNTPGEYTVTLNAINDRGQKADPV

Lmo0514_EGDe    491  TVVVTVTTSDSGHVDPVP------PTPQEETDQTIIPEPQSETTEDSVKEDQAKDGQAKSEEKADKTSLT
Lmo0514_10403S  491  TVVVTVTTSDSGHVDPVPPTPEIKPTPQEETDQTIIPEPQSETTEDSVKEDQAKDGQAKSEEKADKTSLT
Lmo0514_4423    491  TVVVTVTTSDSGHVNPVPPTPEVKPTPQEETDQTIIPEAQSETREDPVKEDQAKAGQAK-----------
Lmo0514_AB27    491  TVVVTVTTSDSGHVNPVPPTPEVKPTPQEETDQTIIPEAQSETREDPVKEDQAKAGQAKSEEKADKTSLT
Lmo0514_F2365   491  TVVVTVTTSDGEHVNPVPPTPEVKPTPQEETDPTIIPEPQSENSEDQVKEN-----NAKTEEKANKTSLT
Lmo0514_ScottA  491  TVVVTVTTSDGEHVNPVPPTPEVKPTPQEETDPTIIPEPQSENSEDQVKEN-----NAKTEEKANKTSLT

Lmo0514_EGDe    555  TKENKVE---KSEKTNQPKTKALPQTGDTNKTALPIAGVMLSLAALLIFRKSKS
Lmo0514_10403S  561  TKENKVE---KSEKTNQPKTKALPQTGDTNKTALPIAGVMLSLAALLIFRKSKS
Lmo0514_4423         ------------------------------------------------------
Lmo0514_AB27    561  MKENKVE---KSEKTNQPKTKALPQTGDTNKTALPIAGVMLSLAALLIFRKSKS
Lmo0514_F2365   556  TKENKVEKADKAEKTNQPKTKALPQTGDTNKSTLPIAGVMLSLAALLIFRKSKS
Lmo0514_ScottA  556  TKENKVEKADKAEKTNQPKTKALPQTGDTNKSTLPIAGVMLSLAALLIFRKSKS
```

(G) Lmo2396

```
Lmo2396_4423     1 MQKKIKIMLVLFLMTTLLLPFSNARAASTDVVNIPDPYLNEGLKNIIGNPFLTELTEANLETITIADISY
Lmo2396_F2365    1 MQKAIKIMLVLFLMTTVFLPFSNVRAASTDVVNIPDPYLNEGLKSIVGNPFLTELTEANLETISVADISY
Lmo2396_ScottA   1 MQKAIKIMLVLFLMTTVFLPFSNVRAASTDVVNIPDPYLNEGLKSIVGNPFLTELTEANLETISVADISY
Lmo2396_EGDe     1 MYKLVKIILALFLITTVFLPFSNVKAAPTDVVNIPDPVLNSGLKNIIGNPFLDEITEANMATITVADLSN
Lmo2396_10403S   1 MYKLVKIILALFLITTVFLPFSNVKAAPTDVVNIPDPVLNSGLKNIIGNPFLDEITEANMATITVADLSN

Lmo2396_4423    71 MYSSPGYPVNGLIKDLTGLEKAVNTTKLYFSNQTEITNLNQIKNLPNLKKIVGITTGLNDIKALSEMPAL
Lmo2396_F2365   71 MNGVPGYAVTGLISDLTGLEKAVNMTKLYFSNQTEIKNLNQIKDLPNLKKIVGVTTGLNDIKALGEMPAL
Lmo2396_ScottA  71 MNGVPGYAVTGLISDLTGLEKAVNMTKLYFSNQTEIKNLNQIKDLPNLKKIVGVTTGLNDIKALGEMPAL
Lmo2396_EGDe    71 MSGAPGYPVTGLIKDLTGLDKAVNMTKLYFSNQSQIKNLDKIKNLPNLKKIVAVTTGLNNISALGEMPAL
Lmo2396_10403S  71 MSGAPGYPVTGLIKDLTGLDKAVNMTKLYFSNQSQIKNLDKIKNLPNLKKIVAVTTGLNNISALGEMPAL

Lmo2396_4423   141 EEVELGGDYITDFTPLLEKENLKSFSYNSYAWLDPAYHQINNEEFEKFANLKSLENLDVTWNNITDLSAL
Lmo2396_F2365  141 EELELGGDYITDFTPLLEKDNLKSFSYNSYAWLNPAYHQIDNEEFKKFTNLKSLESLDVTWNNITDLSSL
Lmo2396_ScottA 141 EELELGGDYITDFTPLLEKDNLKSFSYNSYAWLNPAYHQIDNEEFKKFTNLKSLESLDVTWNNITDLSSL
Lmo2396_EGDe   141 EEVELGGDYITDFTPLLEKENLTSFSYNSYAWSNPAYHQINNDEFKNFTKLKSLVKLDLTWNNITDLSPL
Lmo2396_10403S 141 EEVELGGDYITDFTPLLEKENLTSFSYNSYAWSNPAYHQINNDEFKNFTKLKSLVKLDLTWNNITDLSPL

Lmo2396_4423   211 TANDHITNLNLSFNKFTNVAPIATMKKLKVLYLNNNNLTSIDSLNTLRGLSIAYADNNNITDLSKLKDFF
Lmo2396_F2365  211 TANDHITNLNLSYNKFTNIAPIATMKELKVLYLNNNNLTSIDSLNTLRGLTIAYADNNNITDLSNLKDFF
Lmo2396_ScottA 211 TANDHITNLNLSYNKFTNIAPIATMKELKVLYLNNNNLTSIDSLNTLRGLTIAYADNNNITDLSNLKDFF
Lmo2396_EGDe   211 TENDHITNLNLNYNQFTNVAPIATMKNLKVLYLNNNNLTSIDALNTLRGLTIAYADNNNITDLSNLKNFF
Lmo2396_10403S 211 TENDHITNLNLNYNQFTNVAPIATMKNLKVLYLNNNNLTSIDALNTLRGLTIAYADNNNITDLSNLKNFF

Lmo2396_4423   281 EGMDVVGDYKGLQVNSQTITLPTINIKEGATAISNNPTLDIDGKEMPISSISDGGTVSADNKTVSFSNLP
Lmo2396_F2365  281 EGMDVVGDYKGLQVNNQTITLPTINIKEGGTAISNNPTLDIDGEKMPVSSISDGGTVSTDNKTVSFTNLP
Lmo2396_ScottA 281 EGMDVVGDYKGLQVNNQTITLPTINIKEGGTAISNNPTLDIDGEKMPVSSISDGGTVSTDNKTVSFTNLP
Lmo2396_EGDe   281 EAMVAQGDYEGLQINNQTITLPTINIKKGATANSTNPTLDINGQKMPVSNISNDGTVSADNKTVSFANLP
Lmo2396_10403S 281 EAMVAQGDYEGLQINNQTITLPTINIKKGATANSTNPTLDINGQKMPVSNISNDGTVSADNKTVSFANLP

Lmo2396_4423   351 IGTKTVTYNATFTATSAKGVPLSYSLKVSQPITVSEKTNSSVNIFYKDENGDELATSETISGKSGENYQT
Lmo2396_F2365  351 VGNKTVTYKATFTATSIKGVPLSYSIKVSQPINVSAQSDSTVNVFYKDENGDELAPSETISGKSGENYQT
Lmo2396_ScottA 351 VGNKTVTYKATFTATSIKGVPLSYSIKVSQPINVSAQSDSTVNVFYKDENGDELAPSETISGKSGENYQT
Lmo2396_EGDe   351 IGNKTVTYKAKFTATSSKGVPLSYSINVSQPINVSEQTDSTVSVFYQDENGNELAPTETLSGKSGEDYQT
Lmo2396_10403S 351 IGNKTVTYKAKFTATSSKGVPLSYSINVSQPINVSEQTDSTVSVFYQDENGNELAPTETLSGKSGEDYQT

Lmo2396_4423   421 TEKTITNYKLKEIEGPPSGQFGDSDTTVTYVYEKADGAPVTVKYVDGDGNELATSDTLNGKIDAPYQSTA
Lmo2396_F2365  421 IEKTITNYTLKEIEGQPSGQFGDSDAIVTYVYEKADGAPVTVKYVDADGNELATSDTLNGKIDAPYQTTA
Lmo2396_ScottA 421 IEKTITNYTLKEIEGQPSGQFGDSDATVTYVYEKADGAPVTVKYVDADGNELATSDTLNGKIDAPYQTNP
Lmo2396_EGDe   421 TEKTIANYQLKEIEGQASGQFTDIDSTVTYVYEKADGAPVTVKYVDADGNDLATSDTLNGKIDAPYQTSA
Lmo2396_10403S 421 TEKTIANYQLKEIEGQASGQFTDIDSTVTYVYEKADGAPVTVKYVDADGNDLATSDTLNGKIDAPYQTSA

Lmo2396_4423   491 KSITGWTVKTTPTNANGVFTNTNQTVTYIYEKADGALVTVKYLDGDGNELATSDTLNGKIDAPYQSTAKS
Lmo2396_F2365  491 KSLSGWAVKTTPTNATGVFTNANQTVTYVYEKADGAPVTVKYVDVDGNELATSDTLNGKIDAPYQTTAKS
Lmo2396_ScottA 491 KSLSGWAVKTTPANATGVFTNANQTVTYVYEKADGAPVTVKYVDVDGNELATSDTLNGKIDATYQTTAKS
Lmo2396_EGDe   491 KSLSGWTVKTTPNNATGVFTNSKQTVTYVYEKADGAPVTVKYVDGDGNELATSDTLNGKIDAPYQTTAKS
Lmo2396_10403S 491 KSISDWAVKTTPNNATGVFTNSKQTVTYVYEKADGAPVTVKYVDADGNELATSDTLNGKIDAPYQTSAKS

Lmo2396_4423   561 ITDWTVKTTPANANGVFTNTXQTVTXXYEKADGAXVTVKYLDGDGNELATSDTLNGKIDAPYQSTAKSLS
Lmo2396_F2365  561 LSGWTVKTTPTNATGVFTNANQTVTYVYEKADGAPVTVKYVDADGNELATSDTLNGKIDAPYQSTAKSIT
Lmo2396_ScottA 561 LSGWTVKTTPTNATGVFTNANQTVTYVYEKADGAPVTVKYVDADGNELATSDTLNGKIDAPYQSTAKSIT
Lmo2396_EGDe   561 LSGWTVKTTPNNATGVFTNSKQTVTYVYEKADGAPVTVKYVDADGNELATSDTLNGKIDAPYQTTAKSLS
Lmo2396_10403S 561 LSGWTVKTTPNNATGVFTNSKQTVTYVYEKADGAPVTVKYVDADGNELATSDTLNGKIDAPYQTSAKSLS

Lmo2396_4423   631 GWVVKTTPANANGVFTNTNQTVTYVYEKADGAPVTVKYLDG-----------------------------
Lmo2396_F2365  631 GWTVKTTPANATGVFTNANQTVTYVYEKADGAPVTVKY--------------------------------
Lmo2396_ScottA 631 GWTVKTTPANATGVFTNANQTVTYVYEKADGAPVTVKY--------------------------------
Lmo2396_EGDe   631 GWTVKTTPNNATGVFTNSKQTVTYVYEKADGAPVTVKYVDADGNELATSDTLNGKIDAPYQTTAESLSGW
Lmo2396_10403S 631 GWTVKTTPNNATGVFTNSKQTVTYVYEKADGAPVTVKYVDA-----------------------------
```

```
Lmo2396_4423     672 ---------------------------------------DGNELATSDTLNGKIDAPYQTSAESLSGWTV
Lmo2396_F2365    669 ----------------------------------------------------------------------
Lmo2396_ScottA   669 ----------------------------------------------------------------------
Lmo2396_EGDe     701 TVKTTPNNATGVFTNSKQTVTYVYEKADGAPVTVKYVDGDGNELATSDTLNGKIDAPYQTTAKSLSGWTV
Lmo2396_10403S   672 ---------------------------------------DGNELATSDTLNGKIDAPYQTTAKSLSGWTV

Lmo2396_4423     703 KTTPNNATGVFTNSKQTVTYVYEKADGAPVTVKYVDADGNELATPDTLNGKLDNSYAVTAKNLSGWKLTA
Lmo2396_F2365    669 ----------------------------------VDADGNELATPDTLNGKLDTSYAATAKNLSGWKLTA
Lmo2396_ScottA   669 ----------------------------------VDADGNELATPDT-----------------------
Lmo2396_EGDe     771 KTTPNNATGVFTNSKQTVTYVYEKADGAPVTVKYVDADGNELATPDTLNGKLDTSYAVTAKNLSGWKLTA
Lmo2396_10403S   703 KTTPNNATGVFTNSKQTVTYVYEKADGAPVTVKYVDADGNELATPDTLNGKLDTSYAVTAKNLSGWKLTA
```

(H) P60

```
P60_4423      1 --MKKATIAATAGIAVTAFAAPTIASASTVVVEAGDTLWGIAQSKGTTVDAIKKANNLTTDKIVPGQKLQ
P60_EGDe      1 --MKKATIAATAGIAVTAFAAPTIASASTVVVEAGDTLWGIAQSKGTTVDAIKKANNLTTDKIVPGQKLQ
P60_10403S    1 --MKKATIAATAGIAVTAFAAPTIASASTVVVEAGDTLWGIAQSKGTTVDAIKKANNLTTDKIVPGQKLQ
P60_ScottA    1 MNMKKATIAATAGIAVTAFAAPTIASASTVVVEAGDTLWGIAQSKGTTVDAIKKANNLTTDKIVPGQKLQ
P60_F2365     1 MNMKKATIAATAGIAVTAFAAPTIASASTVVVEAGDTLWGIAQSKGTTVDAIKKANNLTTDKIVPGQKLQ

P60_4423     69 VNNEVAAAEKTEKSVSATWLNVRSGAGVDNSIITSIKGGTKVTVETTESNGWHKITYNDGETGFVNGKYL
P60_EGDe     69 VNNEVAAAEKTEKSVSATWLNVRSGAGVDNSIITSIKGGTKVTVETTESNGWHKITYNDGKTGFVNGKYL
P60_10403S   69 VNNEVAAAEKTEKSVSATWLNVRTGAGVDNSIITSIKGGTKVTVETTESNGWHKITYNDGKTGFVNGKYL
P60_ScottA   71 V-NEVAAAEKTEKSVSATWLNVRSGAGVDNSIITSIKGGTKVTVESTESNGWNKITYNDGETGFVNGKYL
P60_F2365    71 V-NEVAAAEKTEKSVSATWLNVRSGAGVDNSIITSIKGGTKVTVESTESNGWNKITYNDGETGFVNGKYL

P60_4423    139 TDKAVSTPVAPTQEVKKETTTQQAAPAAETKTEVKQTTQATTPAPKVAETKETPVVDQNATTHAVKSGDT
P60_EGDe    139 TDKAVSTPVAPTQEVKKETTTQQAAPAAETKTEVKQTTQATTPAPKVAETKETPVVDQNATTHAVKSGDT
P60_10403S  139 TDKAVSTPVAPTQEVKKETTTQQAAPVAETKTEVKQTTQATTPAPKVAETKETPVIDQNATTHAVKSGDT
P60_ScottA  140 TDKVASTPVAPTQEVKKETTTQQAAPAAETKTEVKQTTQATTPAPKVAETKETPVVDQNATTHAVKSGDT
P60_F2365   140 TDKVASTPVAPTQEVKKETTIQQAAPAAETKTEVKQTTQATTPAPKVAETKETPVVDQNATTHAVKSGDT

P60_4423    209 IWALSVKYGVSVQDIMSWNNLSSSSIYVGQKLAIKQTANTATPKAEVKTEAPAAEKQAAPVVKENTNTNT
P60_EGDe    209 IWALSVKYGVSVQDIMSWNNLSSSSIYVGQKLAIKQTANTATPKAEVKTEAPAAEKQAAPVVKENTNTNT
P60_10403S  209 IWALSVKYGVSVQDIMSWNNLSSSSIYVGQKLAIKQTANTATPKAEVKTEAPAAEKQAAPVVKENTNTNT
P60_ScottA  210 IWALSVKYGVSVQDIMSWNNLSSSSIYVGQKLAIKQTANTATPKAEVKKEAPAAEKQAAPVVKENTNTNT
P60_F2365   210 IWALSVKYGVSVQDIMSWNNLSSSSIYVGQKLAIKQTANTVTPKAEVKKEAPAAEKQAAPVVKENTNTNT

P60_4423    279 ATTEKKETATQQQTAPKAPTEAAKPAPAPSTNTNANK------TNTNTNNTNTNTPSKNTNTNSNTNTNT
P60_EGDe    279 ATTEKKETATQQQTAPKAPTEAAKPAPAPSTNTNANKTNTNTNTNTNTNNTNTNTPSKNTNTNSNTNTNT
P60_10403S  279 ATTEKKETATQQQTAPKAPTEAAKPAPAPSTNTNANK------TNTNTNTNNTNTPSKNTNTNSNTNTNT
P60_ScottA  280 NATTEKKETTQQQTAPKAPTEAAKPAPAPSTNTNANK------TNTNTNNTNTSTPSKNTNTNTNSNTNT
P60_F2365   280 NTTTEKKETTQQQTAPKAPTEAAKPAPAPSTNTNANK------TNTNTNNTNTSTPSKNTNTNTNSNTNT

P60_4423    343 NSNTNANQGSSNNNSNSSASAIIAEAQKHLGKAYSWGGNGPTTFDCSGYTKYVFAKAGISLPRTSGAQYA
P60_EGDe    349 NSNTNANQGSSNNNSNSSASAIIAEAQKHLGKAYSWGGNGPTTFDCSGYTKYVFAKAGISLPRTSGAQYA
P60_10403S  343 NSNTNANQGSSNNNSNSSASAIIAEAQKHLGKAYSWGGNGPTTFDCSGYTKYVFAKAGISLPRTSGAQYA
P60_ScottA  344 NSNTNANQGSSNNNSNSSASAIIAEAQKHLGKAYSWGGNGPTTFDCSGYTKYVFAKAGISLPRTSGAQYA
P60_F2365   344 NSNTNANQGSSNNNSNSSASAIIAEAQKHLGKAYSWGGNGPTTFDCSGYTKYVFAKAGISLPRTSGAQYA

P60_4423    413 STTRISESQAKPGDLVFFDYGSGISHVGIYVGNGQMINAQDNGVKYDNIHGSGWGKYLVGFGRV
P60_EGDe    419 STTRISESQAKPGDLVFFDYGSGISHVGIYVGNGQMINAQDNGVKYDNIHGSGWGKYLVGFGRV
P60_10403S  413 STTRISESQAKPGDLVFFDYGSGISHVGIYVGNGQMINAQDNGVKYDNIHGSGWGKYLVGFGRV
P60_ScottA  414 STTRISESQAKPGDLVFFDYGSGISHVGIYVGNGQMINAQDNGVKYDNIHGSGWGKYLVGFGRV
P60_F2365   414 STTRISESQAKPGDLVFFDYGSGISHVGIYVGNGQMINAQDNGVKYDNIHGSGWGKYLVGFGRV
```

(I) LntA

```
LntA_4423      1  MKEGKRVKKLVAWFNGLSKIWKVVVIIGTVFVVIIALTTGEDESEQTKTKTSSNKIVKTASKPKFSTKDL
LntA_ScottA    1  MKEGKRVKKLVAWFNGLSKMWKVVVIIGAVFVVIIALTTGENEGEQTKTKTNSDKIVKTTSKPKLSTKDL
LntA_F2365     1  -------------------MWKVVVIIGAVFVVIIALTTGENEGEQTKTKTNSDKIVKTTSKPKLSTKDL
LntA_EGDe      1  ------MKKLVAWFNGLSKMWKVVVIIGAVFVVIIALTTGEDEGEQTKTKKDSNKVVKTASRPKLSTKDL
LntA_10403S    1  MKEGKRVKKLVAWFNGLSKMWKVVVIIGAVFVVIIALTTGEDEGEQTKTKKDSNKVVKTASRPKLSTKDL

LntA_4423     71  ALIKADLAEFEGRELSSEKILKDTIKEESWSDLDFANDNINQMIGTMKRYQQEILNIDAVKRSSEASADT
LntA_ScottA   71  ALIKADLAEFEGRELSSEKILKDTIKEESWSDLDFANDNINQMIDTMKRYQQEILSIDAVKRSSEASADT
LntA_F2365    52  ALIKADLAEFEGRELSSEKILKDTIKEESWSDLDFANDNINQMIDTMKRYQQEILSIDAIKRSSEASADT
LntA_EGDe     65  ALIKADLAEFEARELSSEKILKDTIKEESWSDLDFANDNINQMIGTMKRYQQEILSIDAIKRSSEASADT
LntA_10403S   71  ALIKADLAEFEARELSSEKILKDTIKEESWSGLDFANDNINQMIGTMKRYQQEILNIDAIKRSSEASADT

LntA_4423    141  KAFKKVFKEWSDFKIERIQVTIDLLNGKKDSEAAFKKSYPNQIIFKKVRTNKLQTALNNLKVGYALLDSQ
LntA_ScottA  141  EAFKKVFKEWSDFKIERIQVTIDLLNGKKDSEAAFKKSYPNQIIFKKVRTNKLQTALNNLKVGYELLDSQ
LntA_F2365   122  EAFKKVFKEWSDFKIERIQVTIDLLNGKKDSEAAFKKSYPNQIIFKKVRTNKLQTALNNLKVGYELLDSQ
LntA_EGDe    135  EAFKKIFKEWSEFKIERIQVTIDLLNGKKDSEAVFKKTYPNQIIFKKVRTNKLQTALNNLKVGYELLDSQ
LntA_10403S  141  QVFKKVFKEWSDFKIERIQVTIDLLNGKKDSEVAFKKSYPNQIIFKKVRTNKLQTALNNLKVGYELLDSQ

LntA_4423    211  K
LntA_ScottA  211  K
LntA_F2365   192  K
LntA_EGDe    205  K
LntA_10403S  211  K
```
