# Supplementary material for: Comparative genomics of human and non-human Listeria monocytogenes sequence type 121 strains
Source: PLoS One. 2017 May 4;12(5):e0176857. doi: 10.1371/journal.pone.0176857 (PMC5417603; doi:10.1371/journal.pone.0176857)

Figure S4: Organization of the WSS/type VII secretion system (ESAT6) in *L. monocytogenes* EGDe and ST121 strains (represented by strain 6179). Homologous genes are shown in the same colour.

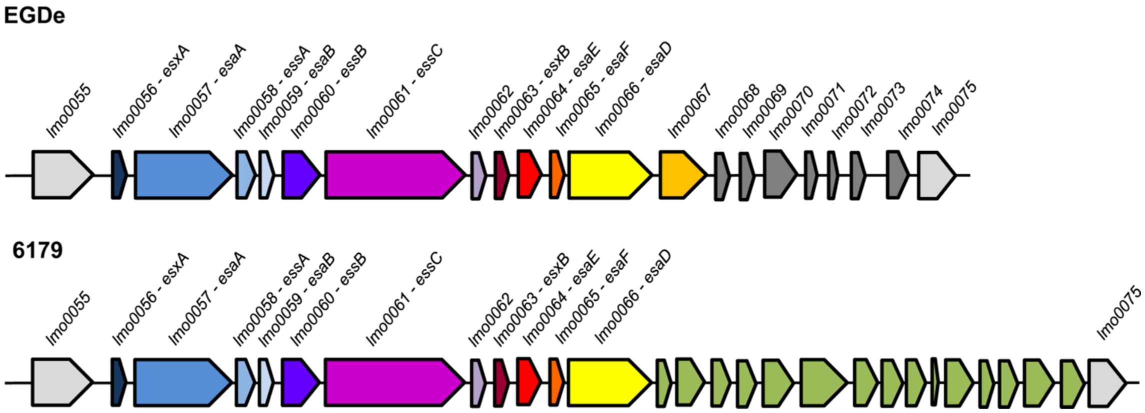

Supplement: S4 Fig — Homologous genes are shown in the same color. (PDF) [file pone.0176857.s004.pdf]
