# Supplementary material for: Comparative genomics of human and non-human Listeria monocytogenes sequence type 121 strains
Source: PLoS One. 2017 May 4;12(5):e0176857. doi: 10.1371/journal.pone.0176857 (PMC5417603; doi:10.1371/journal.pone.0176857)

Fig. S5: Intracellular growth of *L. monocytogenes* ST121 strains in human cell lines. Intracellular growth coefficient (IGC) of three human ST121 isolates (L58-55, N12-0367, N13-0119; all harboring a truncated *inlA*), the food isolate 4423 (truncated *inlA*), and strain P02-001 (food isolate, full length *inlA*) and the reference strains EGDe and ScottA in Caco2 (**A**) and HepG2 cell lines (**B**). Mean values and standard deviations of the three independent biological replicates are presented. Different letters indicate significant differences (P< 0.05) between the IGC of the strains.

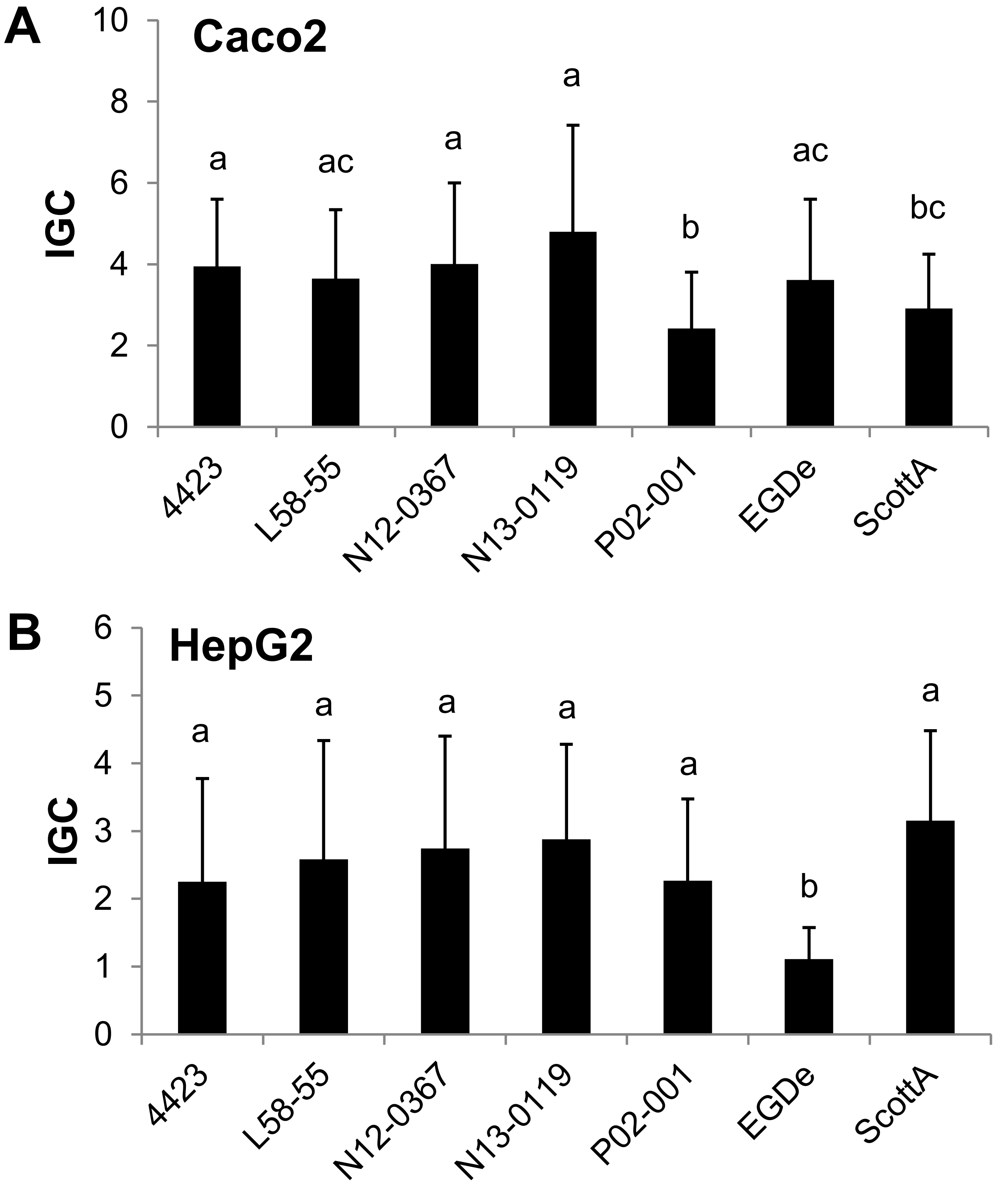

Supplement: S5 Fig — Intracellular growth coefficient (IGC) of three human ST121 isolates (L58-55, N12-0367, N13-0119; all harboring a truncated inlA), the food isolate 4423 (truncated inlA), and strain P02-001 (food isolate, full length inlA) and the reference strains EGDe and ScottA in Caco2 (A) and HepG2 cell lines (B). Mean values and standard deviations of the three independent biological replicates are presented. Different letters indicate significant differences (P< 0.05) between the IGC of the strains. (PDF) [file pone.0176857.s005.pdf]
